# The Protein Neddylation Inhibitor MLN4924 Suppresses Patient-Derived Glioblastoma Cells via Inhibition of ERK and AKT Signaling

**DOI:** 10.3390/cancers11121849

**Published:** 2019-11-22

**Authors:** Suji Han, Hyemi Shin, Jeong-Woo Oh, Yun Jeong Oh, Nam-Gu Her, Do-Hyun Nam

**Affiliations:** 1 Institute for Refractory Cancer Research, Samsung Medical Center, Seoul 06351, Korea; hanbonoboss@gmail.com (S.H.); shinhm0427@gmail.com (H.S.); dhwjddn88@gmail.com (J.-W.O.); yuunzung12@gmail.com (Y.J.O.); 2 Department of Health Sciences & Technology, Samsung Advanced Institute for Health Science & Technology (SAIHST), Sungkyunkwan University, Seoul 06351, Korea; 3 Department of Neurosurgery, Samsung Medical Center, Sungkyunkwan University, Seoul 06351, Korea

**Keywords:** MLN4924, NEDD8, neddylation, glioblastoma

## Abstract

Glioblastoma is a highly aggressive and lethal brain tumor, with limited treatment options. Abnormal activation of the neddylation pathway is observed in glioblastoma, and the NEDD8-activating enzyme (NAE) inhibitor, MLN4924, was previously shown to be effective in glioblastoma cell line models. However, its effect has not been tested in patient-derived glioblastoma stem cells. We first analyzed public data to determine whether NEDD8 pathway proteins are important in glioblastoma development and patient survival. NAE1 and UBA3 levels increased in glioblastoma patients; high NEDD8 levels were associated with poor clinical outcomes. Immunohistochemistry results also supported this result. The effects of MLN4924 were evaluated in 4 glioblastoma cell lines and 15 patient-derived glioblastoma stem cells using high content analysis. Glioblastoma cell lines and patient-derived stem cells were highly susceptible to MLN4924, while normal human astrocytes were resistant. In addition, there were various responses in 15 patient-derived glioblastoma stem cells upon MLN4924 treatment. Genomic analyses indicated that MLN4924 sensitive cells exhibited enrichment of Extracellular Signal Regulated Kinase (ERK) and Protein kinase B (AKT, also known as PKB) signaling. We verified that MLN4924 inhibits ERK and AKT phosphorylation in MLN4924 sensitive cells. Our findings suggest that patient-derived glioblastoma stem cells in the context of ERK and AKT activation are sensitive and highly regulated by neddylation inhibition.

## 1. Introduction

Glioblastoma is the most common and malignant brain tumor [1]. Despite past huge efforts, the median survival of glioblastoma patients is still less than 14.6 months [2] because glioblastoma is more resistant to radiation and chemotherapy than other cancers [3]. Furthermore, there are difficulties in delivering medicine during treatment because of the blood-brain barrier (BBB) [4]. Additionally, a lack of understanding of brain neurobiology is one of the factors which has made it more difficult to develop innovative treatments. For years, many researchers have focused on investigating the molecular mechanisms of the development and progression of glioblastoma, yet there has been little progress in treating this disease. Therefore, it is important to identify major anti-glioblastoma targets, and develop more effective therapeutic methods for the treatment of glioblastoma.

Protein degradation has an important role in maintaining homeostasis of cells, and deregulation of the protein degradation process is related to diseases such as cancer [5,6]. The most representative proteolytic process, the ubiquitin-proteasome system (UPS), has been reported to be associated with apoptosis due to cancer cell growth and drug resistance [7]. Neddylation, one of the protein degradation processes, is a type of protein posttranslational modification [8,9]. In this process, the ubiquitin-like protein Nedd8 combines with the lysine residues of other proteins to change their function. The neddylation process is catalyzed through Nedd8-activating enzyme E1, Nedd8-conjugating enzyme E2 and Nedd8-E3 ligases [10,11,12]. The most important substrate of neddylation is Cullin-RING E3 ligase (CRL), which is a multicomponent ubiquitin ligase that regulates the turnover of several proteins with major roles in cancer biology such as p21, p27, and NF-k-B inhibitor alpha [13]. Recently, increasing experimental evidence strongly suggest that the protein neddylation modification process is overexpressed in various human cancers [14,15,16,17]. These findings indicate that inhibition of protein neddylation can be utilized as an important strategy for the treatment of cancer.

In an effort to treat cancer by controlling protein degradation, a drug called bortezomib (also known as Velcade^®^ or PS-341) that targets the ubiquitin-proteasome system (UPS) was developed [18,19]. However, this drug is toxic to patients because it lacks specificity and inhibits overall protein degradation. Studies aiming at finding cancer-specific drugs recently identified through high-throughput screeningMLN4924 [20,21]. MLN4924 (also known as pevonedistat) is a NEDD8-activating enzyme (NAE) inhibitor, which was discovered as the first anticancer agent in this class [22]. MLN4924 binds to active sites of NAE and inhibits the progress of the next enzyme reaction. As a result, cullin neddylation is suppressed, and this causes the accumulation of CRL-substrate. Previous studies have shown that CRL-substrate accumulation due to MLN4924 generates DNA damage, cell cycle arrest, senescence, and apoptosis in the cells of various cancer species [23,24,25,26]. In addition, there is a study regarding glioblastoma that MLN4924 suppresses cancer cell growth in vitro and in vivo. MLN4924 showed good clinical efficacy in phase I/II clinical trials, and has low toxicity [27]. Furthermore, it has a high potential as a treatment option for glioblastoma because it passes through the BBB.

In this study, we evaluated the efficacy of MLN4924 using various patient-derived glioblastoma stem cells through high content analysis. In addition, we identified various drug reactivity in each cell, and analyzed the factors causing this difference using a genome study.

## 2. Results

### 2.1. The Neddylation Pathway is Overactivated in Patient-Derived Glioblastoma Specimens

**Figure 1:**
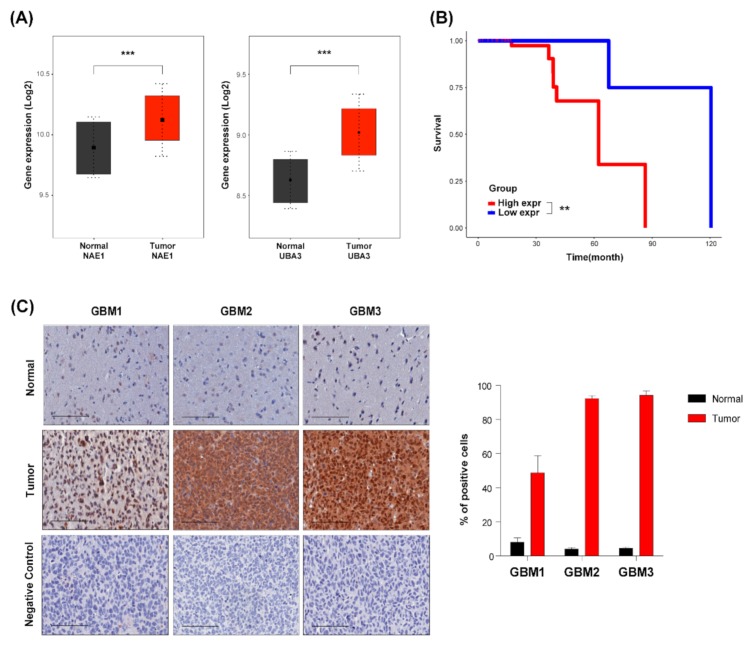
The neddylation pathway is overactivated in patient-derived glioblastoma specimens. (**A**) Gene expression levels that related neddylation pathway across normal brain and glioblastoma specimens on TCGA and Rembrandt data set. (**B**) Kaplan-Meier analysis for survival on a Rembrandt dataset of glioblastoma patients according to their NEDD8 levels. Data analyzed using log-rank (Mantel-Cox) test. (**C**) Immunohistochemical staining of various glioblastoma patient-derived xenograft model specimens using NEDD8-specific antibody. Negative control was stained without primary antibody. Bars represent 100 µm.

Previous studies have shown that the neddylation pathway is overactivated in various types of cancers. Therefore, we first demonstrated whether the neddylation pathway is activated in glioblastoma. Comparing the gene expressions associated with the neddylation pathway in public data including TCGA and Rembrandt, expression levels of the neddylation enzymes NAE1 and UBA3 were higher in glioblastoma than in the normal (Figure 1A). Additionally, a high expression level of NEDD8 is associated with the poor survival of glioblastoma patients (Figure 1B). Kaplan-Meier analysis showed that the overall survival rate was significantly lower in glioblastoma patients with a high expression of NEDD8 compared to patients with a lowNEDD8 expression. Overall survival rate was analyzed using the log-rank (Mantel-Cox) test. Next, we verified the level of NEDD8 protein in glioblastoma using Immunohistochemistry (IHC) staining of Formalin-fixed paraffin-embedded (FFPE) samples from various patient-derived xenograft models. NEDD8 expression was significantly upregulated in glioblastoma compared to the expression level of normal samples (Figure 1C). Quantitative results showed that expression of NEDD8 was about 40-80% higher in glioblastoma than in normal samples (Figure 1C, right). These data indicate that the neddylation pathway was upregulated in glioblastoma, and it can be utilized as a potential therapeutic target.

### 2.2. MLN4924 Inhibits Proliferation and Induces Apoptosis in Glioblastoma Cell Lines

**Figure 2:**
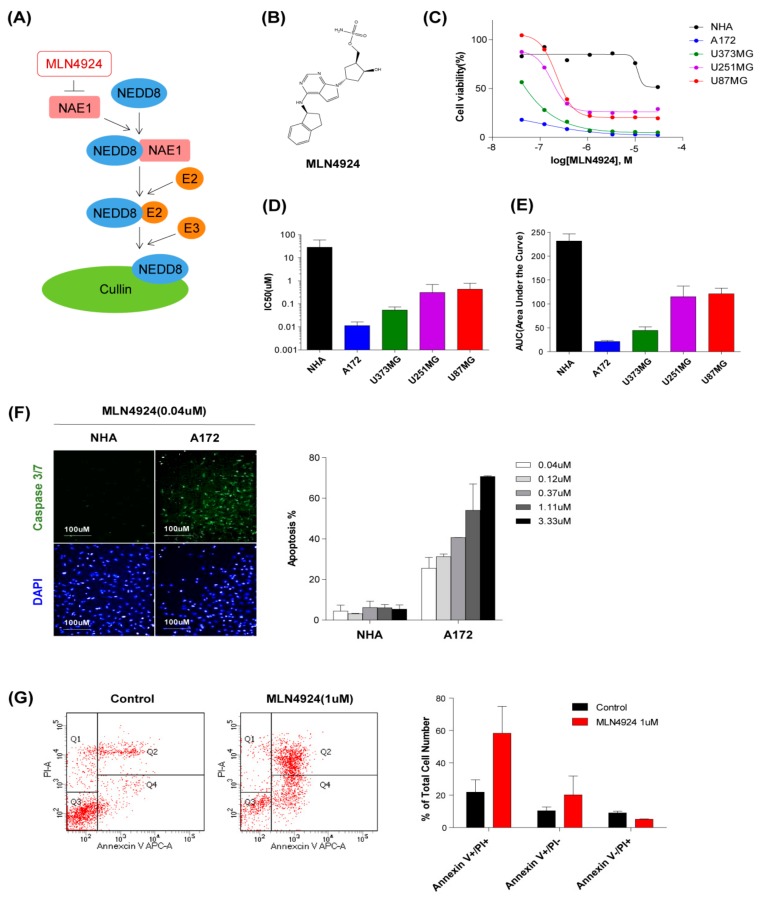
MLN4924 inhibits proliferation and induces apoptosis in glioblastoma cell lines. (**A**) Schematic diagram of the mechanism for MLN4924 inhibiting cullin neddylation. (**B**) Chemical structure of MLN4924. (**C**) Live cells percentage of normal human astrocyte and four cell lines treated with different concentrations (0.04–30 μM) of MLN4924 for 7 d. (**D**) IC_50_ values of MLN4924 in Normal human astrocyte and four cell lines obtained from dose-response curves are shown. (**E**) Area under the curve (AUC) values of MLN4924 in normal human astrocyte and four cell lines obtained from dose-response curves are shown. (**F**) Immunofluorescence staining of Caspase 3/7 induced by MLN4924(0.04 μM) treated cells were demonstrated. Nuclei were labeled with DAPI. Cell images were captured with Operetta High-Content Imaging System and analyzed through Harmony 4.5 software. The error bars represent the standard deviation. (**G**) Flow cytometric analysis of Annexin V-PI stained control (left) or MLN4924 (1 µM, right) treated A172 cells were demonstrated.

We next determined the effect of MLN4924 on four glioblastoma cell lines (A172, U251MG, U373MG, and U87MG), and normal human astrocytes. Cells were treated with various concentrations (0.04–30 μM) of MLN4924 for 7 days. For the quantitative evaluation of drug responses, we used a cell-based screening platform for high content analysis that concurrently measures both cell numbers and biomarker immunofluorescence in 384-well plates. Cell number analyses using the aforementioned assay with increasing concentrations of MLN4924 are shown in Figure 2C. Half-maximal growth-inhibitory concentration (IC_50_) values of MLN4924 in cells were 28.54 μM (NHA), 0.01 μM (A172), 0.31 μM (U251MG), 0.05 μM (U373MG), and 0.43 μM (U87MG), respectively (Figure 2D). Area under the curve values are 231.5 (NHA), 21.35 (A172), 114.9 (U251MG), 44.49 (U373MG), and 121.2 (U87MG), respectively. (Figure 2E). Cell viability decreased due to MLN4924 in all four cell lines, but normal human astrocytes showed a decrease in viability only at the maximum concentration. We also observed that immunofluorescence readouts for Caspase 3/7, which is the apoptosis marker, were increased by MLN4924. When cells were treated with 0.04 μM MLN4924, caspase 3/7 was hardly expressed in normal human astrocytes, whereas A172 cells presented approximately 30% apoptotic cells (Figure 2F). Quantitative results also showed that normal human astrocytes had less than 10% of apoptotic cells, but A172 cells which is the most sensitive to MLN4924 showed dose-dependent ratios of 30~70% (Figure 2F, right). Additionally, the effect of growth inhibition on apoptosis was assessed using flow cytometry and annexin V/PI staining. When A172 cells were treated with MLN4924 (1 μM) for 3 days, the percentage of dead cells increased more than two times compared to those in the control sample after undergoing same treatment (Figure 2G). These results showed a selective inhibitory effect of MLN4924 on the glioblastoma cells.

### 2.3. MLN4924 Suppresses Cullin 1 Neddylation in Patient-Derived Glioblastoma Stem Cells

**Figure 3:**
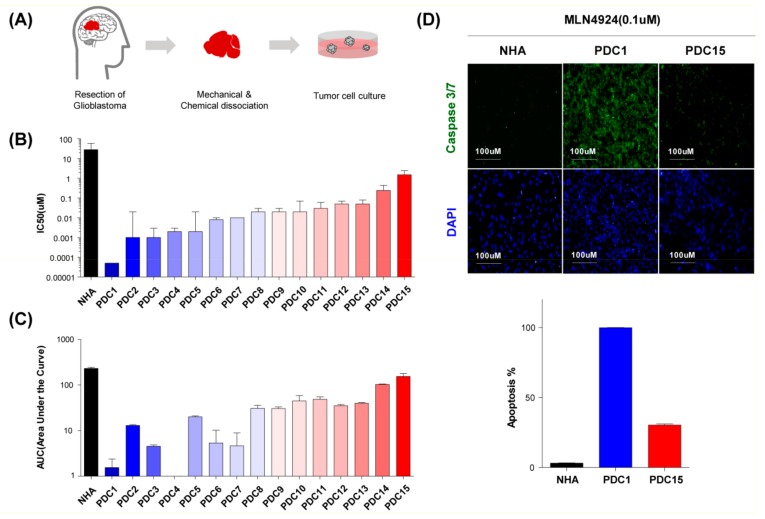

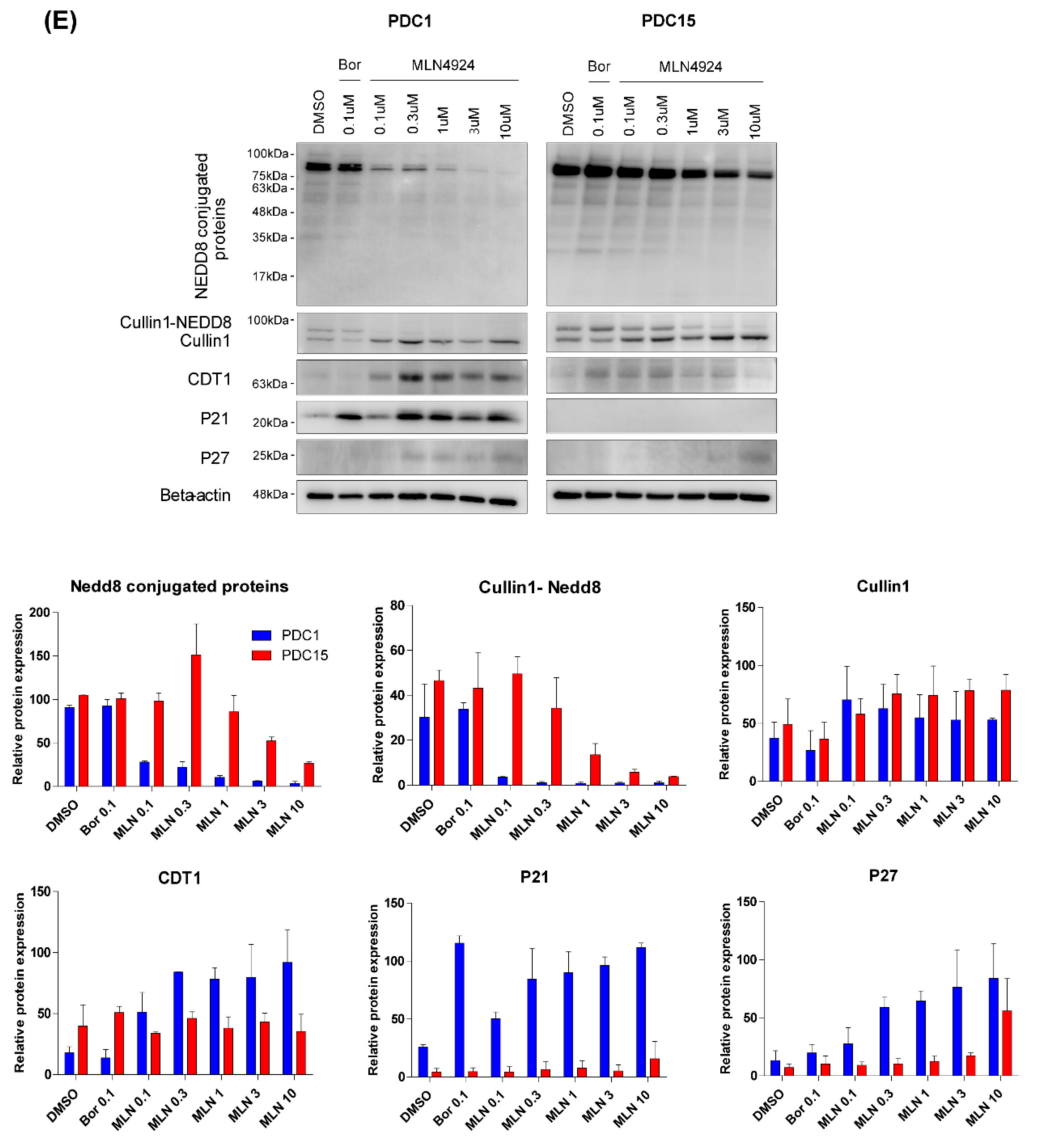
MLN4924 suppress cullin 1 neddylation in patient-derived glioblastoma stem cells. (**A**) Schematic diagram of obtaining PDC from glioblastoma patient tissue. (**B**) IC_50_ values of 15 different patient-derived glioblastoma stem cells treated with different concentrations (0.04–30 μM) of MLN4924 for 7 d. (**C**) AUC values of 15 different patient-derived glioblastoma stem cells treated with different concentrations (0.04–30 μM) of MLN4924 for 7 d. (**D**) Immunofluorescence staining of Caspase 3/7 induced by MLN4924 (0.1 μM for 7 d) treated cells were demonstrated. Nuclei were labeled with DAPI. Cell images were captured with Operetta High-Content Imaging System. (**E**) Cells were treated with MLN4924 and Bortezomib for 12 h at the indicated doses. Neddylation pathway related protein levels were examined by western blotting. Beta actin was used as a loading control. Similar results were obtained in three independent experiments.

We confirmed the growth inhibitory effect of MLN4924 in the cell line, but the conventionally established glioblastoma cell line did not maintain the heterogeneity of glioblastoma [28,29,30,31,32]. Consequently, we constructed patient-derived glioblastoma stem cells to verify the effects of MLN4924. We dissociated 15 patients-derived surgical specimens into single cells, and cultured them in serum-free conditions as in the neurosphere (Figure 3A) [33]. 

We confirmed previously that glioblastoma cells maintain the characteristics of glioblastoma stem cells under these culture conditions [34,35,36]. Cultured cells were seeded on laminin-coated 384-well plates, and treated with increasing concentrations (0.04–30 μM) of MLN4924 for 7 days. The quantitative drug responses were evaluated by high content analysis. Half-maximal growth-inhibitory concentration (IC_50_) values of MLN4924 in patient-derived glioblastoma stem cells are shown in Figure 3B and the area under the curve values are shown in Figure 3C. Patient-derived glioblastoma stem cells generally exhibited a higher sensitivity than normal human astrocytes, and showed various reactivity.

Of the 15 patient-derived glioblastoma stem cells, PDC1 was the most sensitive to MLN4924, while PDC15 was the most resistant. We also observed the immunofluorescence readouts for caspase 3/7 in these cells. When using 0.1 μM MLN4924 for 7 days, we noticed that caspase 3/7 was highly expressed in PDC1 compared to the expression levels in PDC15 (Figure 3D). Quantitative analysis results indicated that PDC1 had more than 90% of apoptotic cells whereas PDC15 showed less than 30% apoptotic cells (Figure 3D, lower). Next, we confirmed the different efficacy of MLN4924 for the inhibition of the neddylation pathway in PDC1 and PDC15 using a western blot assay. Cells were treated with various concentrations (0.1–10 μM) of MLN4924 for 12 h and compared with bortezomib (0.1 μM), which is the pan-proteasome inhibitor. In both cells, global protein expression and cullin neddylation were reduced, however, PDC1 was more sensitive to MLN4924 than PDC15, and showed high efficacy even at low concentrations (Figure 3E).

The expression of CDT1, P21, and P27 was also increased dose-dependently in PDC1, but not in PDC15. In addition, we identified changes in the neddylation pathway in PDC6 and PDC14 that were more resistant to MLN4924 than PDC1, and more sensitive to MLN4924 than PDC15. As confirmed using the western blot assay, PDC6 and PDC14 had less inhibitory effect of neddylation by MLN4924 than PDC1, while it showed higher neddylation inhibition level than PDC15 (Figure S1). Collectively, these data demonstrate that each cell has various responsiveness levels to the neddylation inhibitory effect of MLN4924.

### 2.4. Response of MLN4924 is Related to ERK and AKT Signaling Pathway

**Figure 4:**
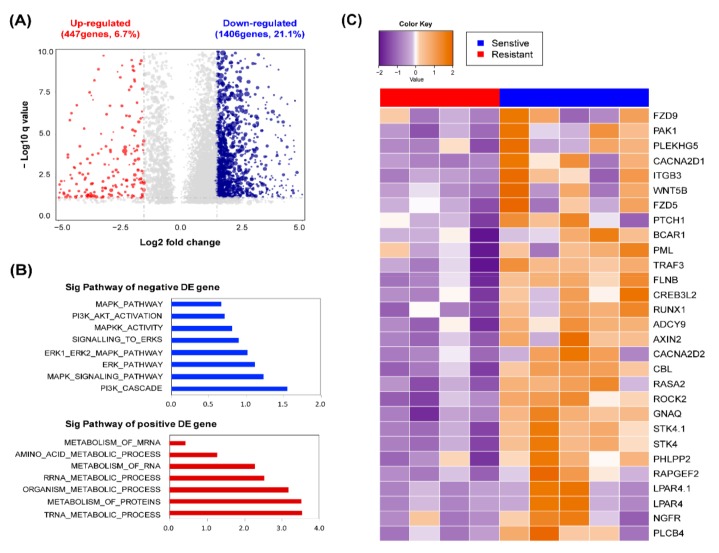
Responses of MLN4924 are related to ERK and AKT signaling pathway. (**A**) Differentially expressed genes betweensensitive and resistance groups are plotted as a volcano plot. The blue dots indicate genes that are enriched, as they are sensitive to MLN4924, while the red dots depict genes that are enriched as resistant to MLN4924. (**B**) Bar charts depict the top ranked pathway analyzed from the functional classification base on GO biological process. Blue (upper) and red (bottom) bars represent negative and positive pathways on the responses of MLN4924. (**C**) Heat map across 9 patient-derived glioblastoma samples which used the top ranked DE genes between the sensitive group and resistant group to MLN4924.

To determine what factors causes a different reactivity to MLN4924 in each cell, we analyzed the genetic information of 15 patient-derived glioblastoma stem cells. First, we divided 15 cells into three groups based on the half-maximal inhibitory concentration (IC_50_) of MLN4924 [37]. Cells were categorized into high, intermediate, and low MLN4924 resistance (Figure S2). Five patient-derived glioblastoma stem cells, PDC1, PCD2, PDC3, PDC4, and PDC5 showed high MLN4924 sensitivity (IC_50_< average (IC_50_) − 1/2 SD (standard deviation)), whereas PDC12, PDC13, PDC14, and PDC15 showed low MLN4924 sensitivity (IC_50_> average (IC_50_) + 1/2 SD). To assess whether specific gene expression signatures predict MLN4924 sensitivity and resistance, we performed transcriptome analysis of five MLN4924 sensitive cells and four resistant cells using RNA sequencing (RNAseq). Cells with an IC_50_ in the mean 1/2 SD were considered intermediates that were not resistant or sensitive and were excepted from the analysis to confirm distinct genetic differences [38,39]. Transcriptome analysis revealed a set of differentially expressed genes between the MLN4924 sensitive group and resistant group (Figure 4A). We observed that 1406 of 6674 genes (21.1%) were significantly enriched in the MLN4924 sensitive group, while another 447 genes (6.7%) were enriched in the MLN4924 resistant group. In addition, we compared the ranking of the pathways enriched by NES in the KEGG, GO, BIOCARTA, and REACTOME genesets. Through the analysis of biological functions of the gene set enriched pathway, we identified that the MLN4924 sensitive group associated with ERK–MAPK and PI3K–AKT pathways, meanwhile the MLN4924 resistant group was involved in the metabolism pathway (Figure 4B, Figure S3). Eight pathways were significantly associated with the MLN4924 sensitive group, and seven pathways related with the resistant group (｜log2 fc｜≥ 1.5, *p*-value ≤ 0.05). Genes related to the ERK-MAPK and PI3K-AKT pathways, which are highly expressed in the MLN4924 sensitive group, are shown in Figure 4C.

### 2.5. Sensitivity of MLN4924 is Associated with Upregulation of ERK and AKT Signaling

**Figure 5:**
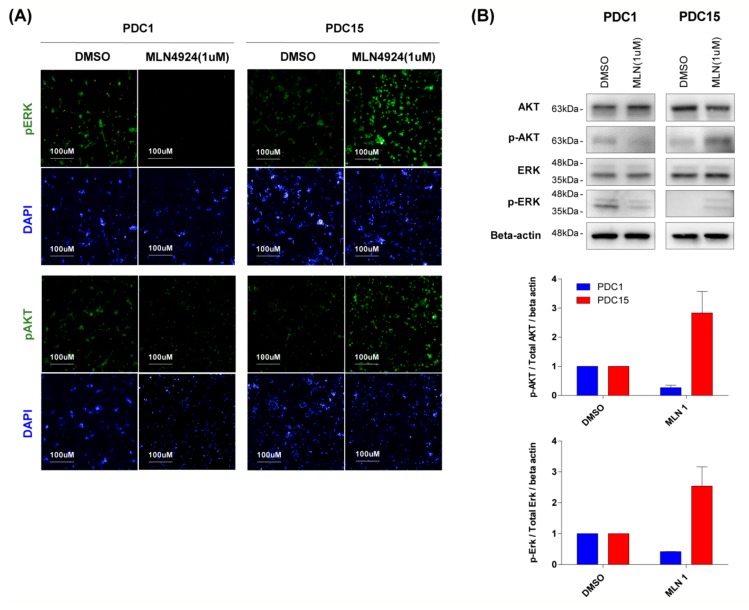
Sensitivity of MLN4924 is associated with upregulation of ERK and AKT signaling. (**A**) Immunofluorescence staining of pERK and pAKT were demonstrated in cells induced by MLN4924 (1 μM) treatment. Nuclei were labeled with DAPI. Cell images were captured with Operetta High-Content Imaging System. (**B**) PDC1 and PDC15 cells were treated with MLN4924 for 12 h at the indicated concentrations. AKT, pAKT, ERK, and pERK levels were examined by western blotting. Beta actin was used as a loading control. Similar results were obtained in three independent experiments.

Analysis of gene expression confirmed that the reactivity of MLN4924 was related to the ERK and AKT pathways. To verify this, we observed changes in ERK and AKT signaling through high content analysis. The cells seeded on the laminin coating plate were treated with MLN4924 (1 μM) for 12 h. The cells were then immunofluorescence-stained with pERK and pAKT specific antibodies. Upon MLN4924 treatment, PDC1, which relies heavily on the ERK and AKT signaling pathway, showed a decrease in ERK and AKT phosphorylation (Figure 5A, left). On the contrary, a MLN4924 resistant cell PDC15 activated a bypass pathway, which increased ERK and AKT phosphorylation (Figure 5A, right). The same tendency was confirmed in western blot results (Figure 5B). When treated with MLN4924, phosphorylation of ERK and AKT decreased in PDC1 but increased in PDC15. Quantitative results showed that treatment with MLN4924 in the cells reduced phosphorylation of ERK and AKT about half in PDC1, but increased 2-3 fold in PDC15 (Figure 5B, lower). These results demonstrated that activation of the ERK and AKT signaling pathway in cells determines reactivity to MLN4924. Additionally, it showed the possibility of predicting MLN4924 efficacy in patients.

## 3. Discussion

Glioblastoma is the most aggressive brain tumor known, and it is highly resistant to radiation and chemotherapy [1,2,3]. In addition, the blood brain barrier (BBB) makes drug delivery difficult, which is one of the reasons most glioblastoma patients have a poor prognosis [4]. Many cancer researchers have recently focused on the protein degradation process and its association with cancer. In general, the protein degradation process in a normal cell degrades unwanted proteins to maintain cell homeostasis. However, in cancer cells, the protein degradation process works abnormally [5,6]. Bortezomib, a drug that targets an abnormal protein degradation process, has been developed, but the lack of specificity for cancer cells has caused many side effects in clinical practice [18,19]. After much research, a cancer-specific drug MLN4924 was identified through high-throughput screening [20,21]. MLN4924 is a drug that inhibits neddylation, which is one of the protein degradation processes [22]. Previous studies have shown that MLN4924 inhibits cell growth and causes apoptosis in various carcinomas [23,24,25,26].

Therefore, in this study, we tested the efficacy of MLN4924 in glioblastoma cells. Experiments of efficacy in normal cells and four glioblastoma cell lines showed that MLN4924 reduced proliferation, and caused apoptosis in cancer cells specifically. Human cancer cell lines have been customarily used in preclinical cancer biology studies. However, the general process of establishing human cell lines causes irreversible loss of important biological properties. Cell lines are homogeneous and undifferentiated compared to actual cancer tissues through prolonged selection pressure under in vitro culture conditions [40].

Accordingly, we extracted the cells by dissociating surgically resected glioblastoma patient-derived cancer tissues to reflect the characteristics of the actual patients. We obtained cells from 15 glioblastoma patients, and treated them with MLN4924 to perform high content analysis. High content analysis is a cell biological research method that integrates image acquisition, process, and analysis. Advances in fluorescence microscopy and image processing technology have made high content analysis an important technology in the field of drug development [41]. We used this technique to evaluate viability, drug responsiveness, and apoptosis of patient-derived glioblastoma stem cells. As with the glioblastoma cell lines, growth of patient-derived glioblastoma stem cells was inhibited and showed cell death by MLN4924. Interestingly, 15 patient-derived glioblastoma stem cells showed different responsiveness to MLN4924. The IC_50_ values of the most sensitive cell and resistant cell differed by more than four-digits. This is a very encouraging result compared to the two-digit difference in IC_50_ values between the most sensitive and resistant cell lines. The most sensitive patient-derived cells and resistant patient-derived cells also showed differences in the expression levels of proteins associated with the neddylation pathway. This means that the patient-derived glioblastoma stem cells reflect the varying drug reactivity of the patients.

Consequently, we performed a genomic study to determine the key factors that cause different reactivity to MLN4924. We analyzed the genes and pathways enriched in these genes that differed significantly between the five sensitive and four resistant cells of MLN4924. As a result, the MLN4924 sensitive group had more active ERK and AKT signaling pathways compared to the resistant group. ERK and AKT signaling pathways are one of the most frequently activated signal transduction pathways in various human cancers. These signals consist of Ras/Raf/MEK/ERK and Ras/PI3K/Prime Time Entertainment Network (PTEN) / Protein kinase B (AKT, also known as PKB) / mammalian target of rapamycin (mTOR) signaling cascades, and are known to play a significant role in regulating cancer cell growth and survival [42,43,44,45]. In addition, increased expression of these signaling pathways can be correlated with sensitivity to targeted therapy compared to patients that do not exhibit elevated expression [43,44,46]. Our findings indicated that the upregulation of ERK and AKT signaling pathways is associated with MLN4924 sensitivity.

This tendency was confirmed not only in genome studies, but also in high content analysis and western blot results. PDC1, which is highly dependent on the ERK and AKT signaling pathways, showed a decrease in ERK and AKT phosphorylation after MLN4924 treatment. On the other hand, PDC15 was not affected by MLN4924 treatment. Rather the bypass pathway was activated, phosphorylation of ERK and AKT was increased. Further studies are required to find a mechanism for bypassing the ERK and AKT signaling pathways in MLN4924 resistant cells. If so, we can overcome drug resistance through a combination treatment with MLN4924 and other target inhibitors.

Although our hypothesis was validated only at the in vitro level, the results using patient-derived glioblastoma stem cells could reflect actual patient characteristics. Our findings can be used to predict the effects of MLN4924 on each glioblastoma patient. In addition, combination therapy utilizing other drugs may further improve the therapeutic effect.

## 4. Materials and Methods 

### 4.1. Cell Lines and Culture Conditions 

Glioblastoma cell lines (A172, U251MG, U373MG, and U87MG) were purchased from the American Type Culture Collection (ATCC, Manassas, VA, USA). All procedures were approved by the Samsung Medical Center (SMC) Institutional Review Board (IRB) and conducted in accordance with the Declaration of Helsinki. Ethical approval code number is 2005-04-001 (approval date: 2005-07-01), 2010-04-004 (approval date: 2010-04-23) and 2013-10-072 (approval date: 2013-10-15). Cells were grown in either Dulbecco's Modified Eagle Medium (11995-065, Gibco, New York, NY, USA) or MEM-alpha (12571-063, Gibco) with 10% fetal bovine serum (16000-044, Gibco) and 1% penicillin-streptomycin (15140-163, Gibco).Normal human astrocytes (CC-2565) were purchased from Lonza (Walkersville, MD USA) and cultured using the AGM Astrocyte growth Bullet kit (CC-3186, Lonza).

### 4.2. Patient-Derived Glioblastoma Specimens and Primary Cell Culture

Surgical specimens were acquired from the glioblastoma patients who had brain tumor removal surgery at the Samsung Medical Center (Seoul, Korea) in consensus with the appropriate Institutional Review Boards. Genomic and molecular information of these tumors were previously reported. Surgically removed tumor specimens were enzymatically dissociated into single cells, following the methods previously reported. Dissociated glioblastoma cells were cultured in neurobasal media (10888-022, Gibco) with N2 supplement (0.5X, 17502-048, Gibco, NY, USA), B27 supplement (0.5X, 12587-010, Gibco), human recombinant basic fibroblast growth factor (bFGF) (20 ng/mL, AF-100-18B, Peprotech, Rocky Hill, NJ, USA), epidermal growth factor (EGF) (20 ng/mL, AF-100-15, Peprotech), 1% penicillin-streptomycin (15140-163, Gibco), and 1% L-glutamine (25030, Gibco) [33]. All samples utilized in this study were used after obtaining written informed consent from patients. This study was approved by the Samsung Medical Center (SMC) Institutional Review Board (IRB) and conducted in accordance with the Declaration of Helsinki on 15th October 2013 (approval number: 2013-10-072).

### 4.3. Drugs

MLN4924 and bortezomib for in vitro studies were purchased from Selleck Chemical (Houston, TX, USA). Stock solutions of each drug (10 mM) were made in 100% dimethyl sulfoxide (DMSO) and stored at −20 °C until use.

### 4.4. Immunofluorescence Staining and High Content Analysis

Patient-derived glioblastoma stem cells (1.5 × 10³) were seeded per well in a laminin-coated 384-well plates, treated with MLN4924 (0.04–30 μM), and incubated at 37 ℃. After 7 days culture medium was removed, and cells were fixed with 4% paraformaldehyde. Cells were then washed with PBS solution, blocked with 1% BSA, and 0.3% triton X-100 in PBS. After blocking, cells were counter-stained with Hoechst 33342 solution. Later they were incubated with primary antibodies overnight at 4 °C, and then with anti-rabbit Alexa 488 (Gibco) secondary antibodies for 1 h with washing in between. Cell images were captured with an Operetta High-Content Imaging System (Perkin Elmer, Waltham, MA, USA), and high content analysis was performed through the Harmony 4.5 software. For evaluating apoptosis, Caspase 3/7 detection reagent was added in cell culture medium (C10423, Thermo Fisher Scientific, Waltham, MA, USA) and the fluorescence intensity per cell was analyzed.

### 4.5. Western Blot Assay

Patient-derived glioblastoma stem cells were lysed in RIPA buffer (20-188, Millipore, Billerica, MA, USA); after which a protease inhibitor (11836153001, Roche, Mannheim, Germany), phosphatase inhibitor (04906837001, Roche, Mannheim, Germany), iodoacetamide (A3221, Sigma Aldrich, St. Louis, MO, USA), and N-ethylmaleimide (23030, Thermo Fisher Scientific) were added. Total proteins (25 μg/lane) were divided by SDS-PAGE, and transferred to PVDF membranes with dry a blotting system (Thermo Fisher Scientific). The membranes were blocked for 1h with 5% bovine serum albumin or 5% skim milk in TBS-T, and incubated with antibodies againstproteins of interest at 4 °C overnight. The following antibodies were used: anti-Nedd8 (2754, Cell Signaling Technology, Danvers, MA, USA), anti-CDT1 (8064, Cell Signaling Technology), anti-Cullin (71-8700, Invitrogen, Waltham, MA, USA), anti-p21 (2947, Cell Signaling Technology), anti-p27 (sc-1641, Santa Cruz Biotechnology, Dallas, TX, USA), anti-beta actin (ab8277, Abcam, Cambridge, UK), anti-AKT (4691, Cell Signaling Technology), anti-P-AKT (4060, Cell Signaling Technology), anti-ERK (9102, Cell Signaling Technology), anti-P-ERK (9101, Cell Signaling Technology), anti-Caspase 3 (9662, Cell Signaling Technology), anti-Cleaved Caspase 3 (9661, Cell Signaling Technology), anti-PARP (9542, Cell Signaling Technology), anti-Cleaved PARP (9541, Cell Signaling Technology), anti-BAX (5023, Cell Signaling Technology), anti-BCL2(2870, Cell Signaling Technology), anti-p53(2524, Cell Signaling Technology), anti-P-p53(S20) (ab157454, Abcam).

### 4.6. Immunohistochemistry

Ten-micrometer-thick sections sliced from paraffin-embedded specimens were prepared on slides. Tumor-containing sections were deparaffinized in xylene, and rehydrated in graded concentrations of ethanol. For antigen retrieval, sections were boiled for 10 min. Remaining peroxidase activity was blocked by incubation with 3% hydrogen peroxide in methanol. Each section was treated with a blocking solution (1% bovine serum albumin in PBS, 0.025% Triton X-100) using the host serum of the secondary antibody. Sections were immunostained with the following antibodies: anti-Nedd8 (2754, Cell Signaling Technology). Biotinylated secondary antibodies (Vector Laboratories, Orton Southgate, UK) were used at a 1:1000 dilution for 1 h, and treated with SignalStain Boost IHC Detection Reagent (8114, Cell Signaling Technology) for 30 min. Diaminobenzidine tetrahydrochloride was used as the enzyme substrate to observe the specific antibody localization, and Harris hematoxylin was used as a nuclear counterstain.

### 4.7. Flow Cytometry-Based Apoptosis Detection

To analyze the apoptotic cell population, patient-derived glioblastoma stem cells were collected in Falcon FACS tubes (352235, Corning Life Sciences, Teterboro, NY, USA), and washed with PBS. Cells were resuspended in Annexin V binding buffer (556454, BD Pharmingen, San Jose, CA, USA), and Annexin V-APC Recombinant Protein (BMS306APC, eBioscience, San Diego, CA, USA) was added. After 30 min, cells were stained with PI (Sigma Aldrich), and analyzed using flow cytometry (FACS Calibur, BD Biosciences, San Jose, CA, USA). The flow cytometry data were interpreted with the Flow Jo TM software (Treestar Inc, Ashland, OR, USA).

### 4.8. RNA Sequencing

RNA sequencing libraries were prepared by using the Illumina TruSeq RNA Library Preparation Kit v2. The sequenced reads were trimmed and mapped onto hg19 using GSNAP version 2012-12-20 [47]. The resulting aligned reads were summarized into BED files using SAMtools and bedTools (bamToBed version 2.16.2) [48]. The BED files were used to estimate reads per kilobase of transcript per million reads (RPKM) using the R package DEGseq [49].

DEGseq: Differentially expressed genes were identified using the R package ‘DEGseq’ [49]. Samples were divided into two groups based on the clinical response to bevacizumab.

Gene ontology(GO) term analysis was performed using a Gene Set Enrichment Analysis (GSEA) with the GenePattern software (http://software.broadinstitute.org/cancer/software/genepattern) for the identification of enriched pathways [50].

RNA sequencing data reported in this paper was uploaded to data repository (The European Genome-phenome Archive (EGA, https://ega-archive.org): Coordinates have been deposited with accession code EGAS00001004018).

### 4.9. Statistical Analysis

Data are presented as means+standard deviation. For group comparisons, the 2-tailed Student’s t-test was conducted using the Graphpad Prism software (version 5.01; GraphPad Software, San Diego, CA, USA) Overall survival curves were plotted according to the Kaplan-Meier method. All differences were considered to be statistically significant at the level of * *p* < 0.05, ** *p* < 0.01, *** *p* < 0.001.

## 5. Conclusions

Our study was the first to identify the effect of a NEDD8-activating enzyme (NAE) inhibitor MLN4924 in patient-derived glioblastoma stem cells. We confirmed that MLN4924 had superior growth inhibition effect in patient-derived glioblastoma cells compared to normal cells. In particular, as ERK and AKT signaling was activated, it was verified to be sensitive to MLN4924.

## Supplementary Materials

The following are available online at https://www.mdpi.com/2072-6694/11/12/1849/s1, Figure S1: Western blot analysis of neddylation pathway-related protein expressions in four different patient-derived glioblastoma stem cells, Figure S2: 15 patient-derived glioblastoma stem cells segregated into sensitive, intermediate, and resistant groups according to their sensitivity to MLN4924, Figure S3: Enrichment plots for indicated gene sets on the responses of MLN4924.
