# Supplementary material for: The Protein Neddylation Inhibitor MLN4924 Suppresses Patient-Derived Glioblastoma Cells via Inhibition of ERK and AKT Signaling
Source: Cancers (Basel). 2019 Nov 22;11(12):1849. doi: 10.3390/cancers11121849 (PMC6966592; doi:10.3390/cancers11121849)
Supplement: Supplementary file 1 [file cancers-11-01849-s001.zip › Supplementary materials.docx]

Supplementary materials


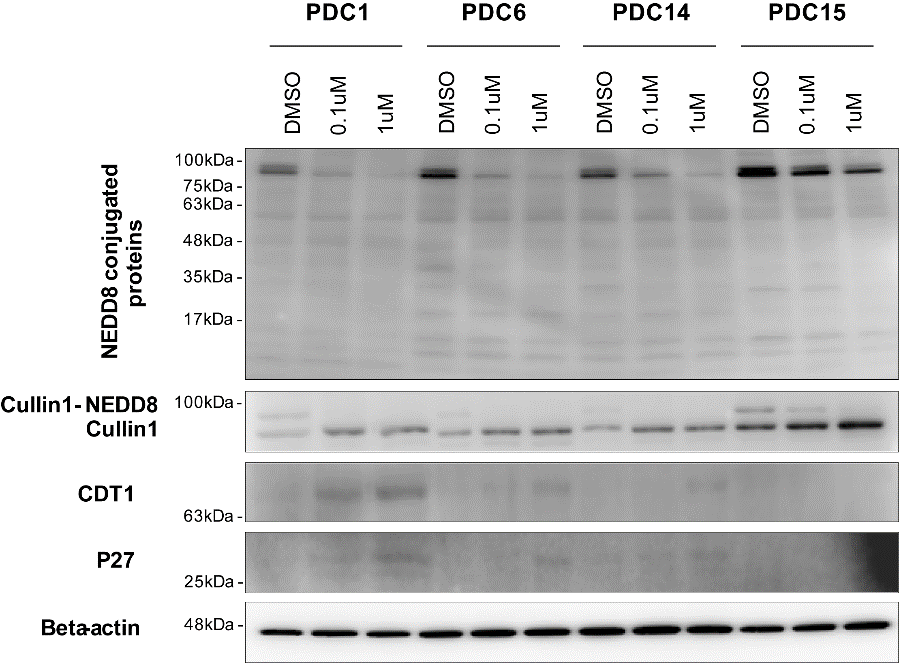


**Figure S1.** Western blot analysis of the neddylation pathway related protein expression in four different patient-derived glioblastoma stem cells.


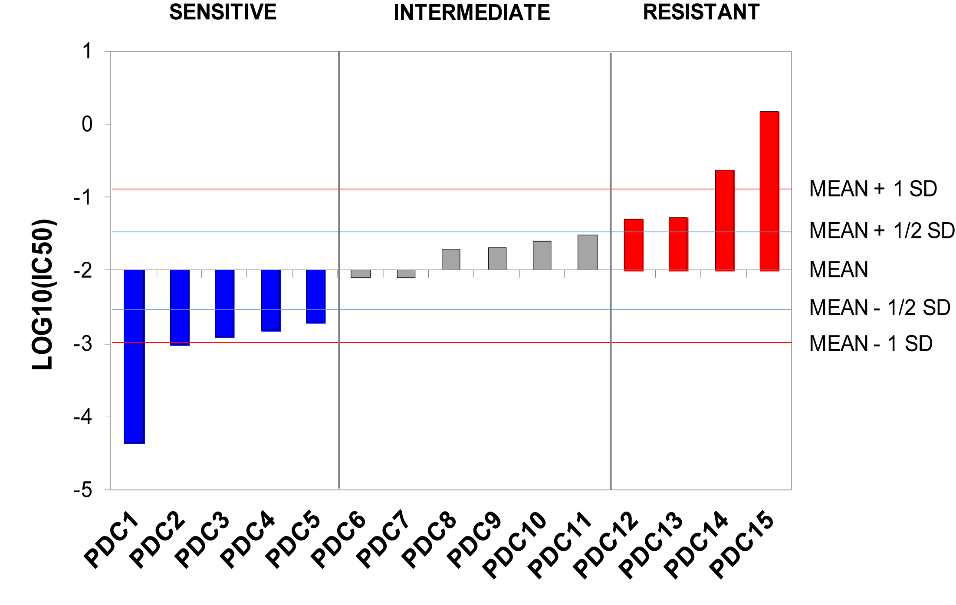


**Figure S2.** Fifteen patient-derived glioblastoma stem cells segregated into sensitive, intermediate, and resistant groups according to their sensitivity to MLN4924. Sensitive cells: IC_50_ < average (IC_50_) − 1/2 SD (standard deviation); resistant cells: IC_50_ > average (IC_50_) + 1/2 SD.


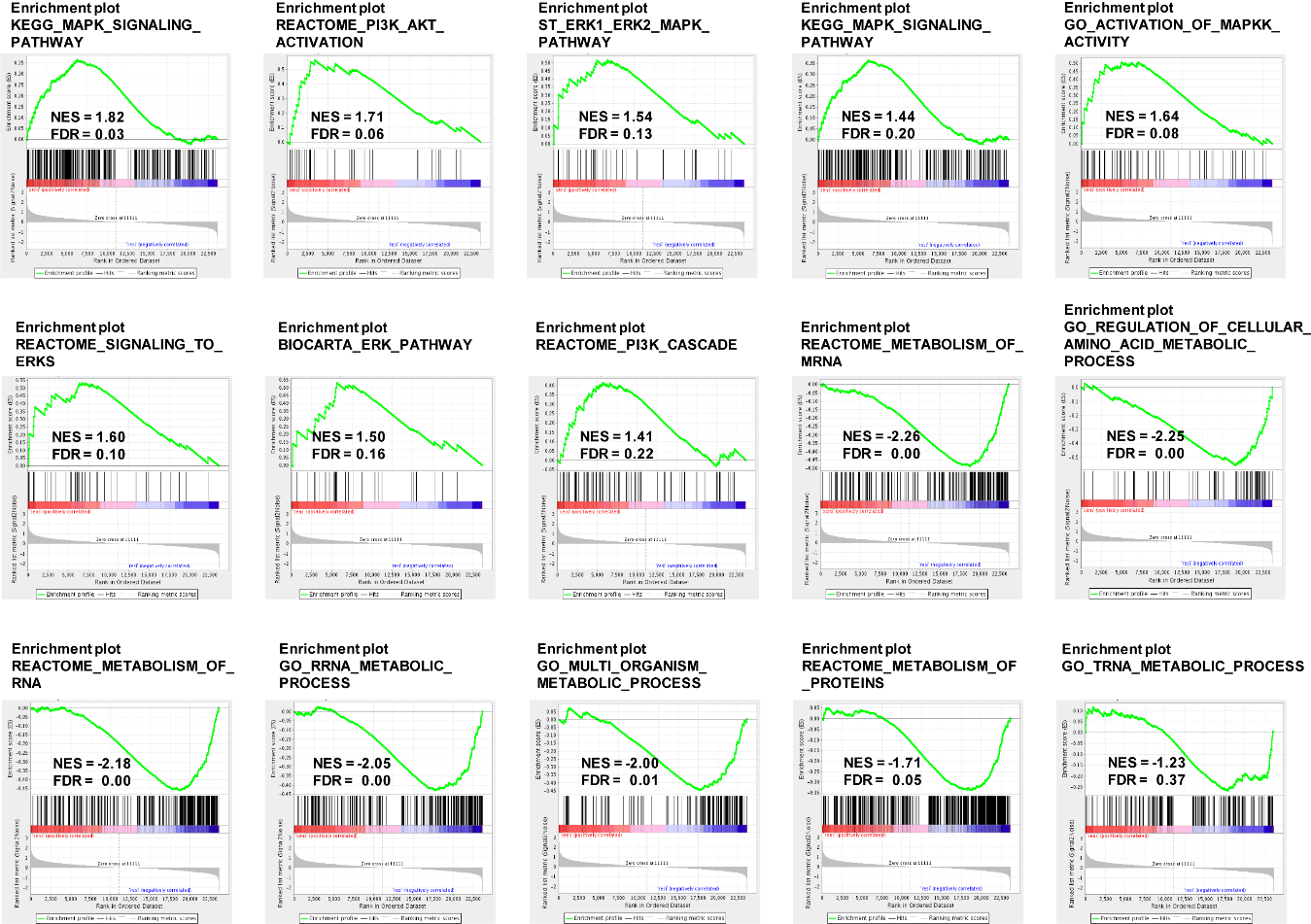


**Figure S3.** Enrichment plots for the indicated gene sets on the responses of MLN4924.
