# Supplementary figures and images for: The Protein Neddylation Inhibitor MLN4924 Suppresses Patient-Derived Glioblastoma Cells via Inhibition of ERK and AKT Signaling
Source: Cancers (Basel). 2019 Nov 22;11(12):1849. doi: 10.3390/cancers11121849 (PMC6966592; doi:10.3390/cancers11121849)

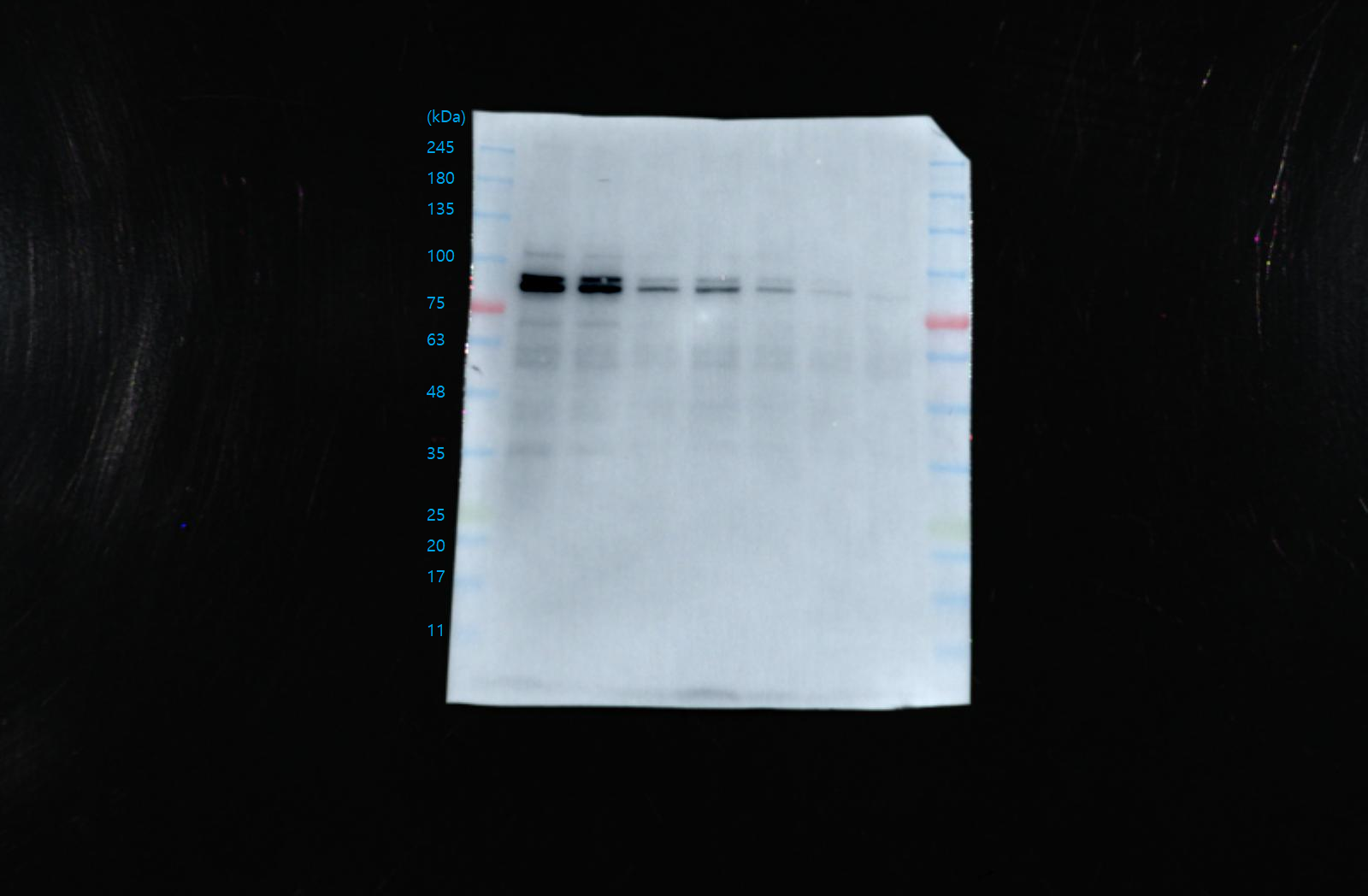

Supplement: Supplementary file 1 [file cancers-11-01849-s001.zip › Figure3(E)_western blot whole blot/PDC1_Nedd8 conjugated proteins.tif]

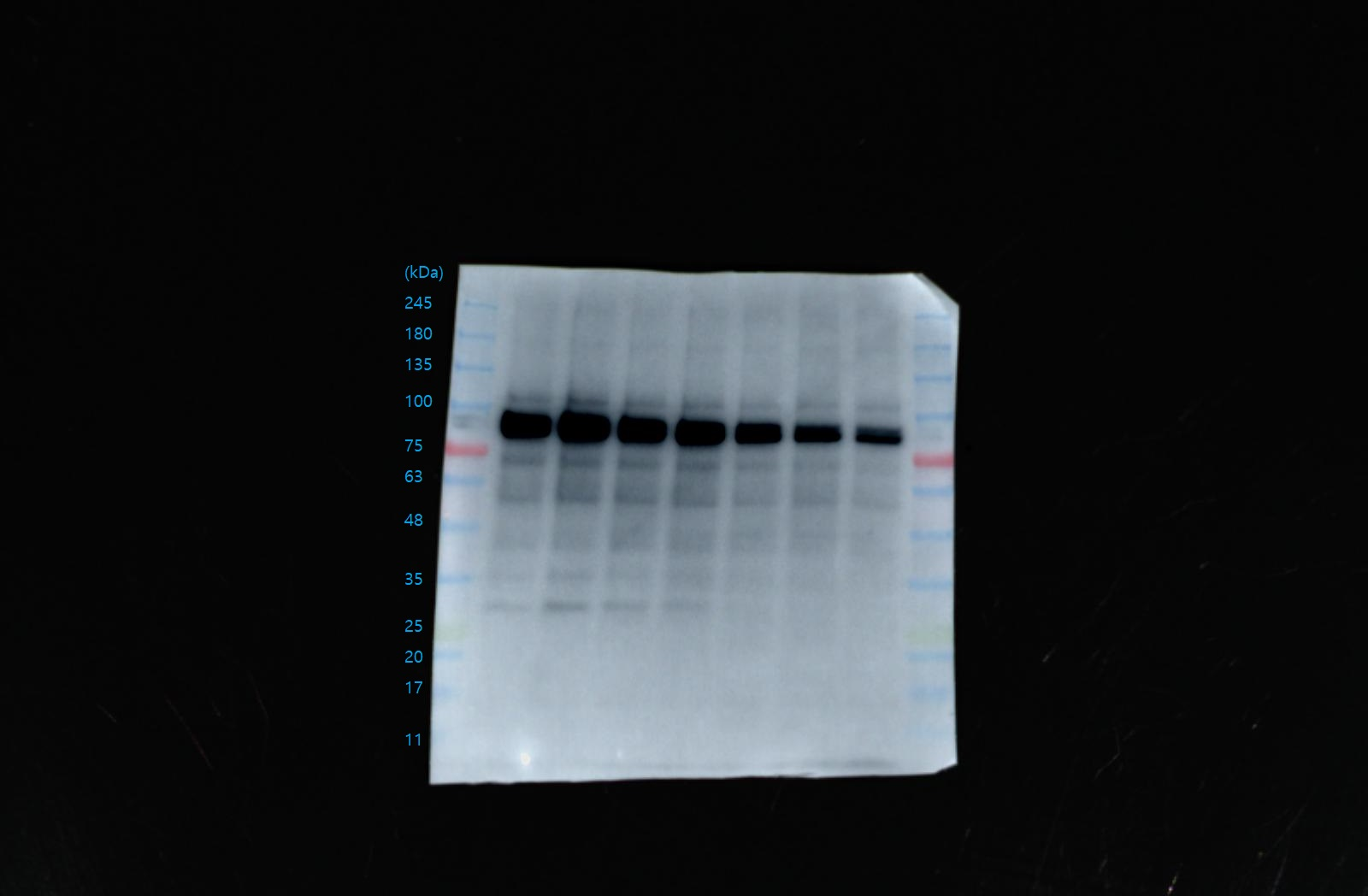

Supplement: Supplementary file 1 [file cancers-11-01849-s001.zip › Figure3(E)_western blot whole blot/PDC15_Nedd8 conjugated proteins.tif]

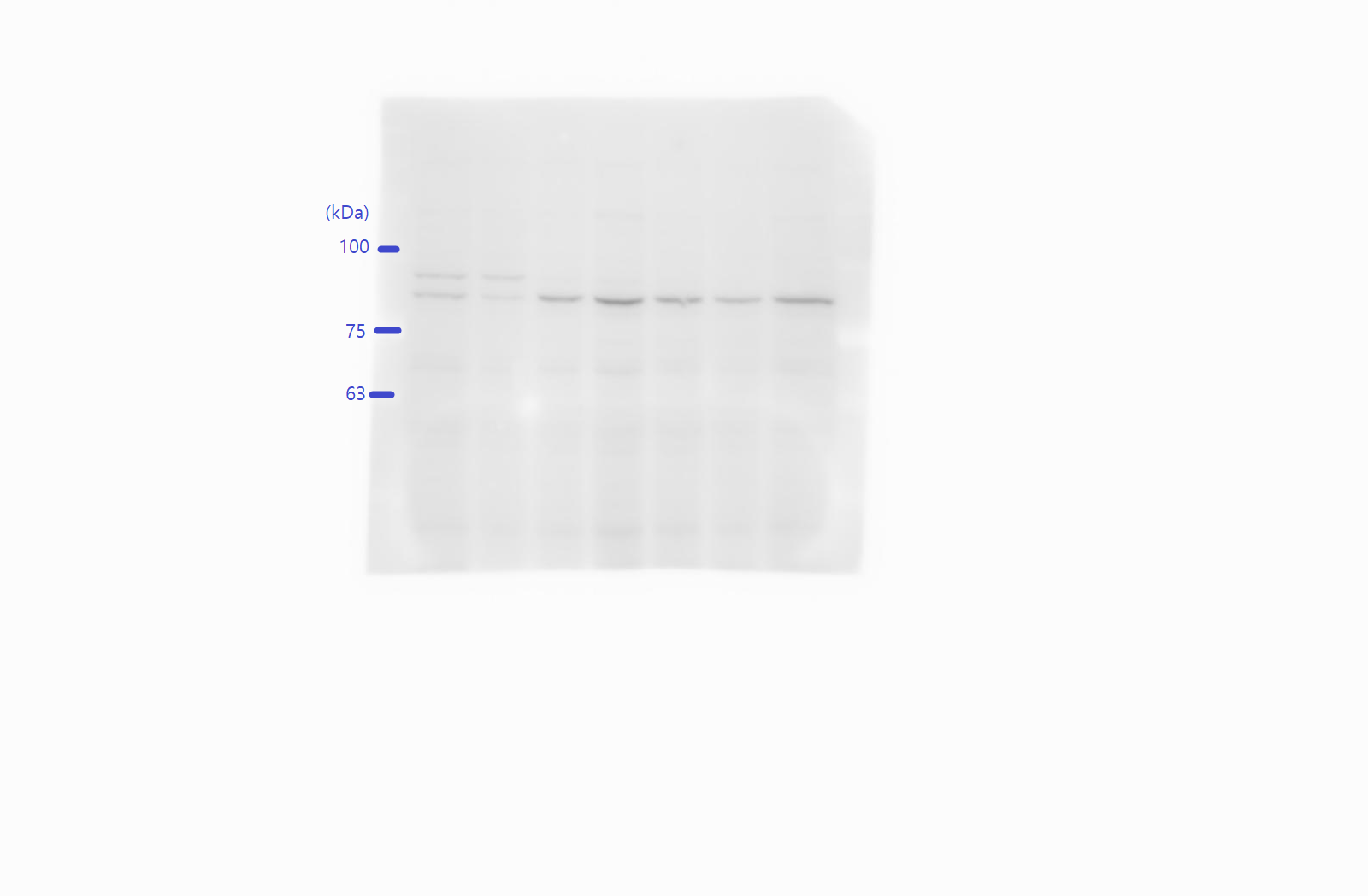

Supplement: Supplementary file 1 [file cancers-11-01849-s001.zip › Figure3(E)_western blot whole blot/PDC1_Cullin1.tif]

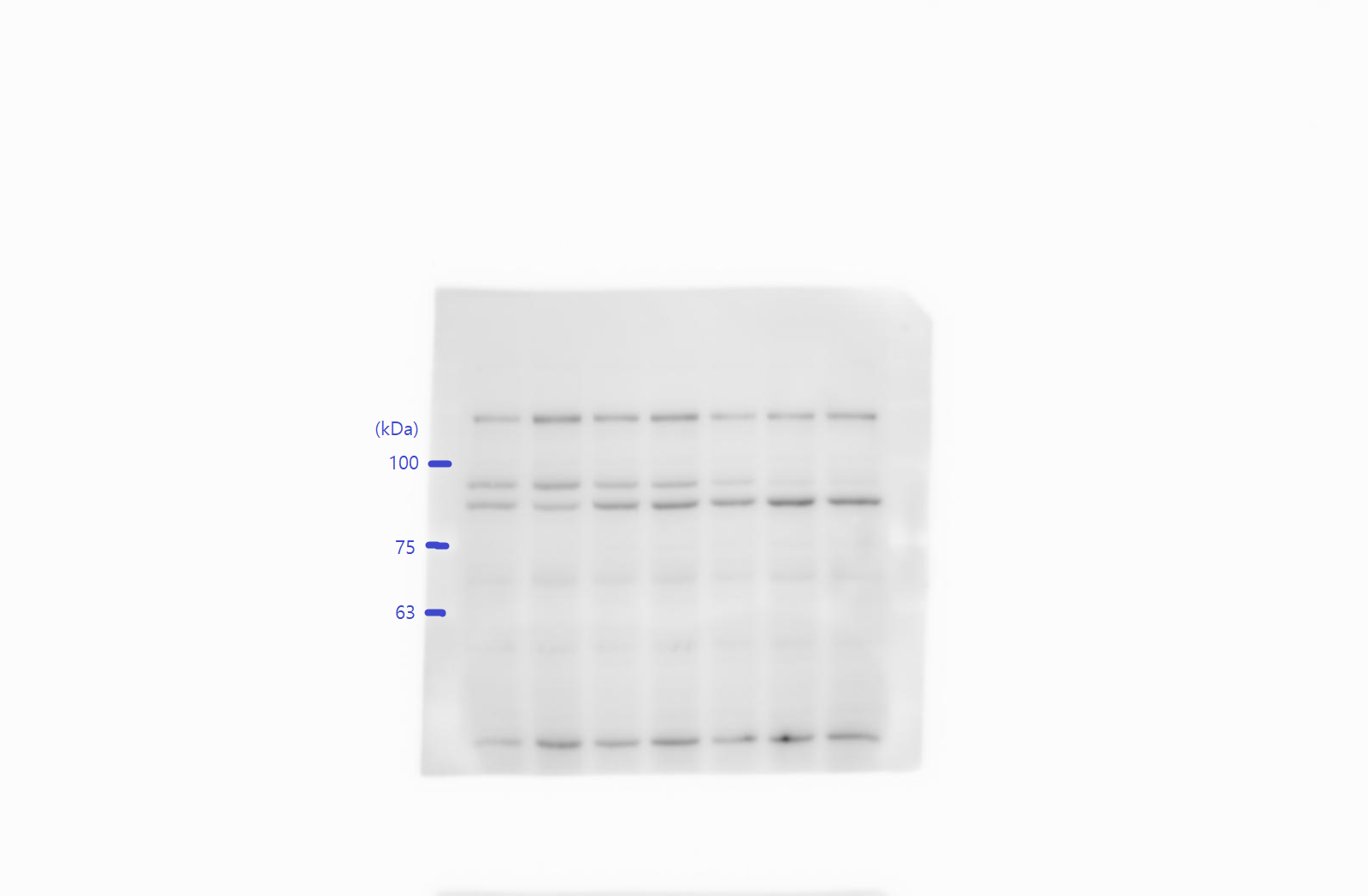

Supplement: Supplementary file 1 [file cancers-11-01849-s001.zip › Figure3(E)_western blot whole blot/PDC15_Cullin1.tif]

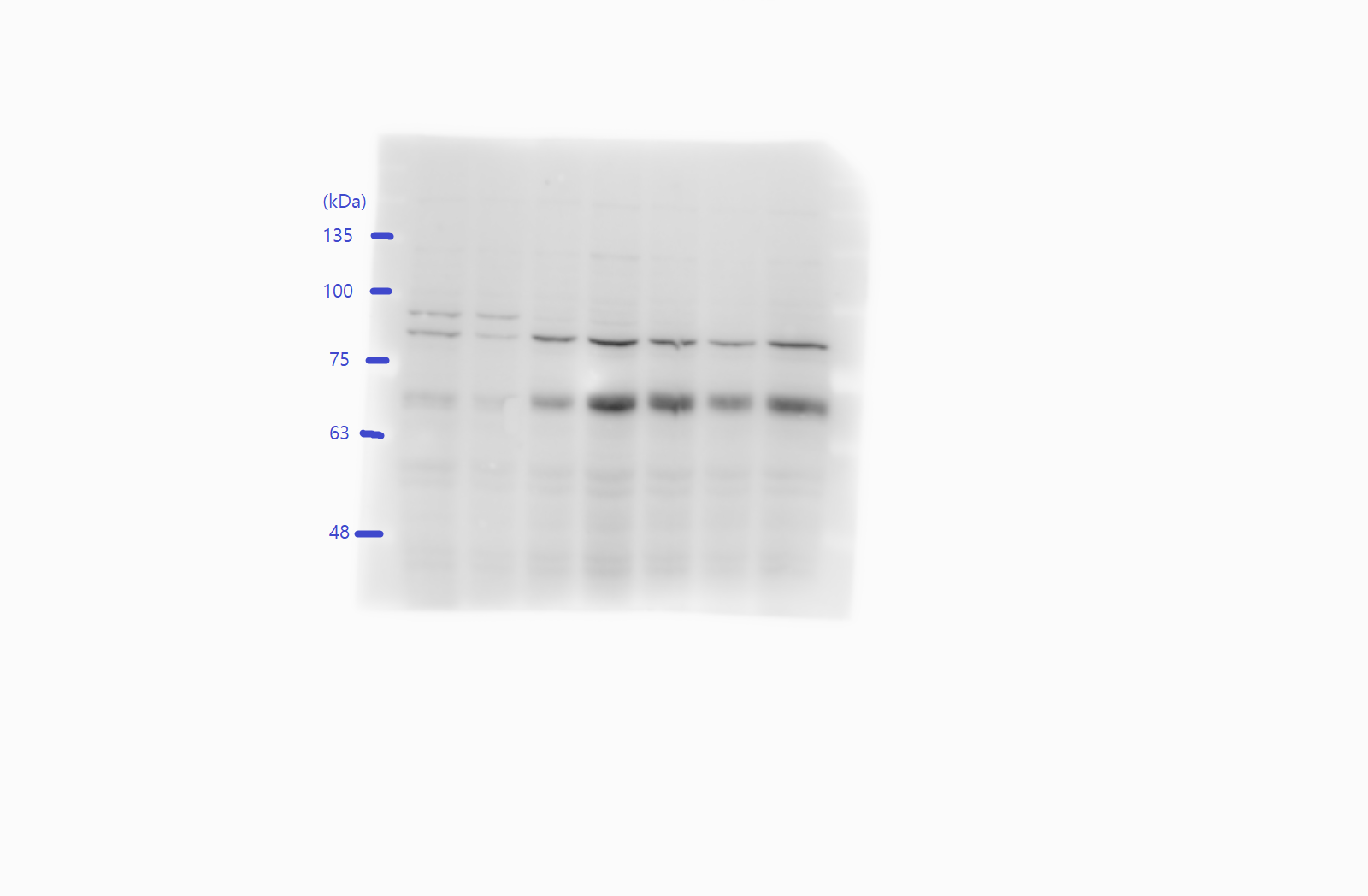

Supplement: Supplementary file 1 [file cancers-11-01849-s001.zip › Figure3(E)_western blot whole blot/PDC1_CDT1.tif]

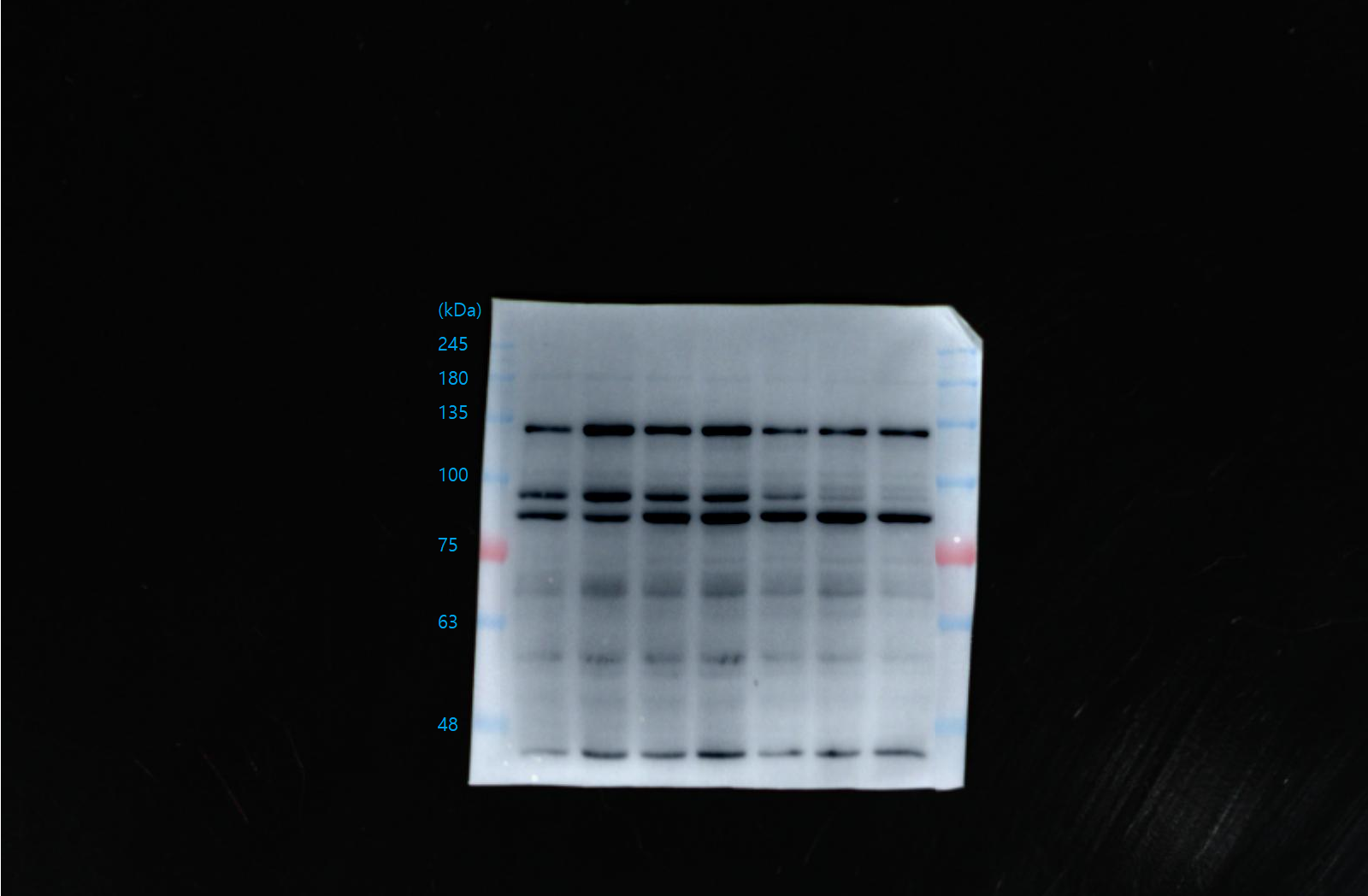

Supplement: Supplementary file 1 [file cancers-11-01849-s001.zip › Figure3(E)_western blot whole blot/PDC15_CDT1.tif]

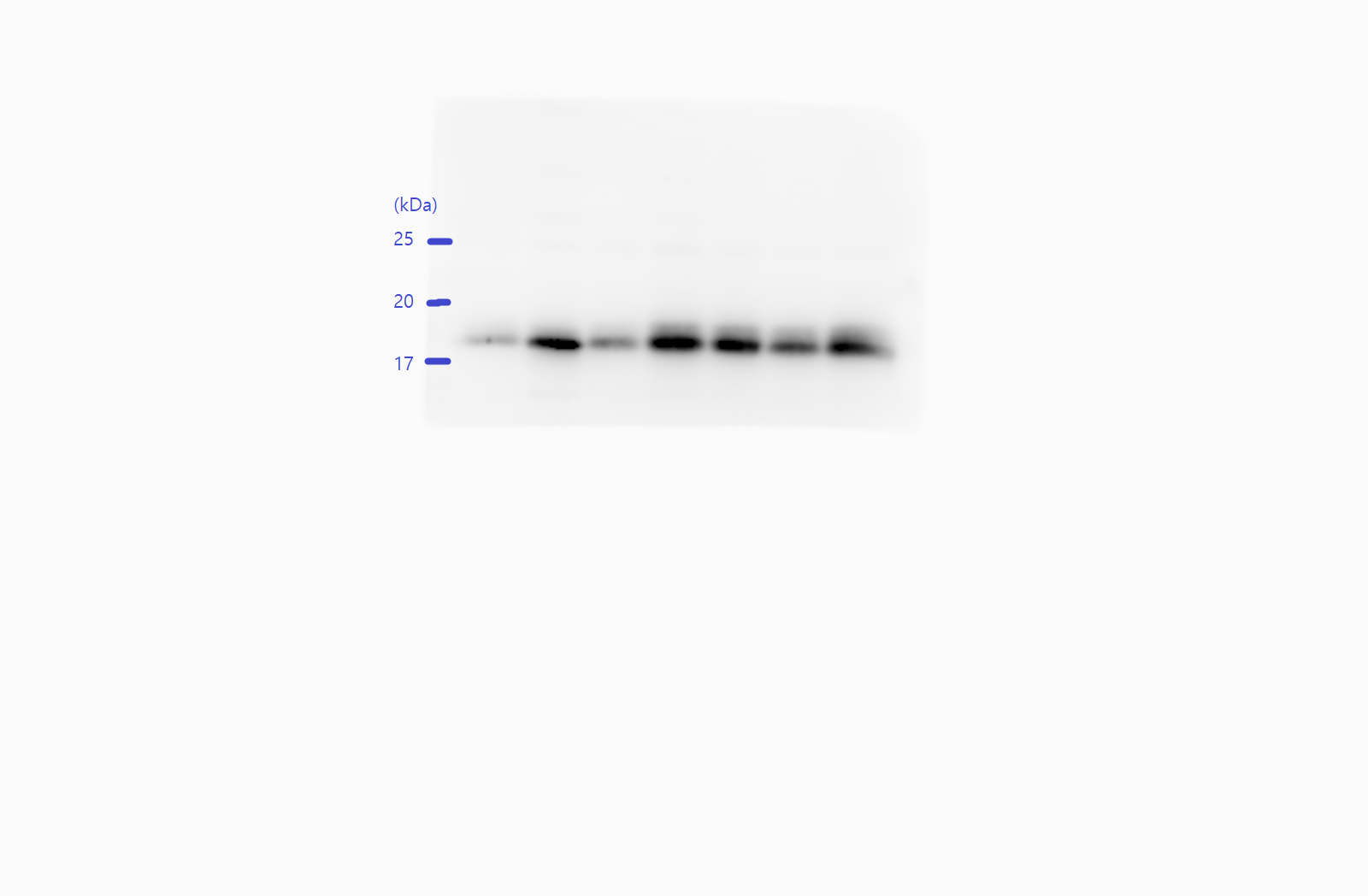

Supplement: Supplementary file 1 [file cancers-11-01849-s001.zip › Figure3(E)_western blot whole blot/PDC1_P21.tif]

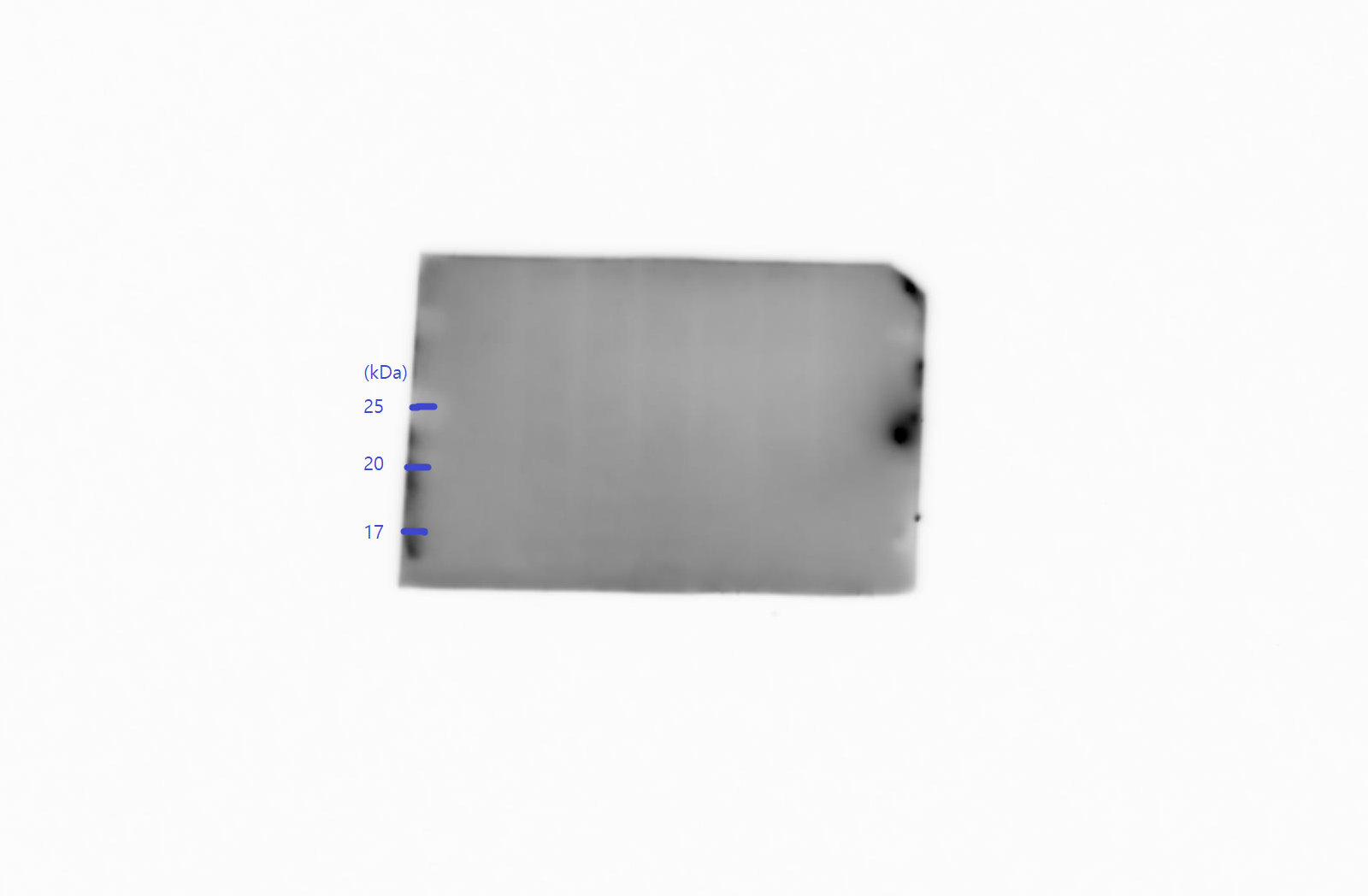

Supplement: Supplementary file 1 [file cancers-11-01849-s001.zip › Figure3(E)_western blot whole blot/PDC15_P21.tif]

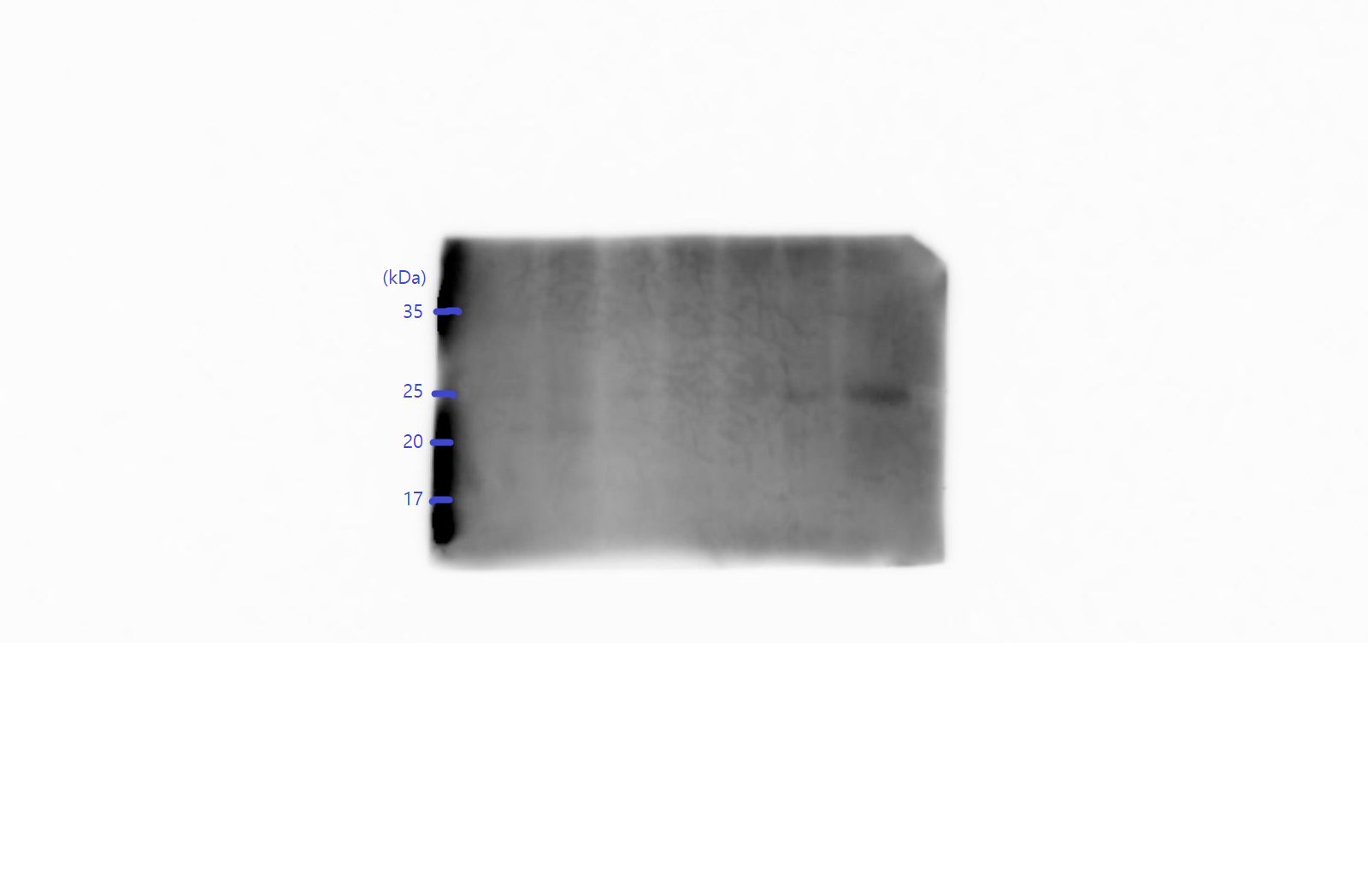

Supplement: Supplementary file 1 [file cancers-11-01849-s001.zip › Figure3(E)_western blot whole blot/PDC15_P27.tif]

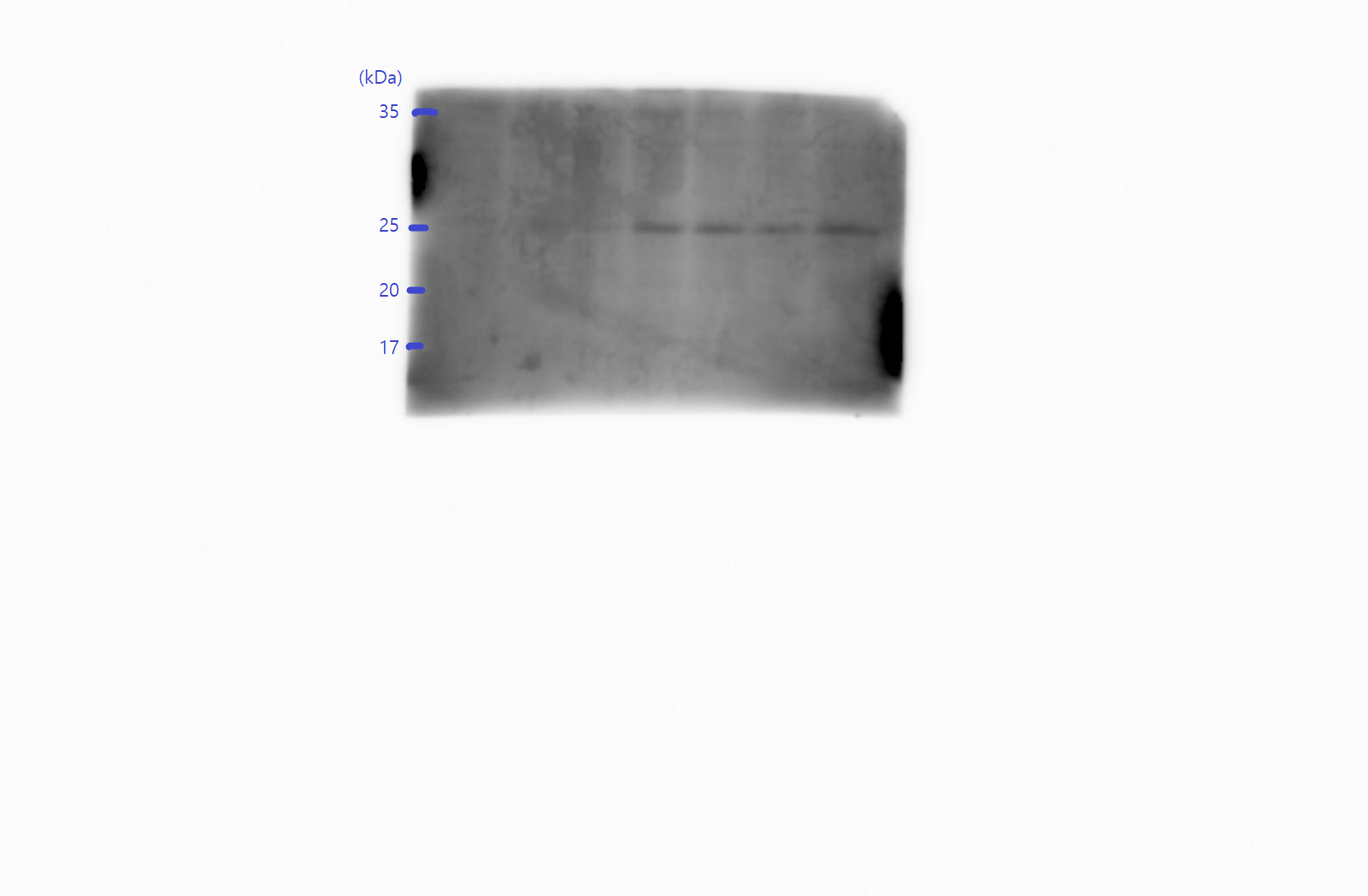

Supplement: Supplementary file 1 [file cancers-11-01849-s001.zip › Figure3(E)_western blot whole blot/PDC1_P27.tif]

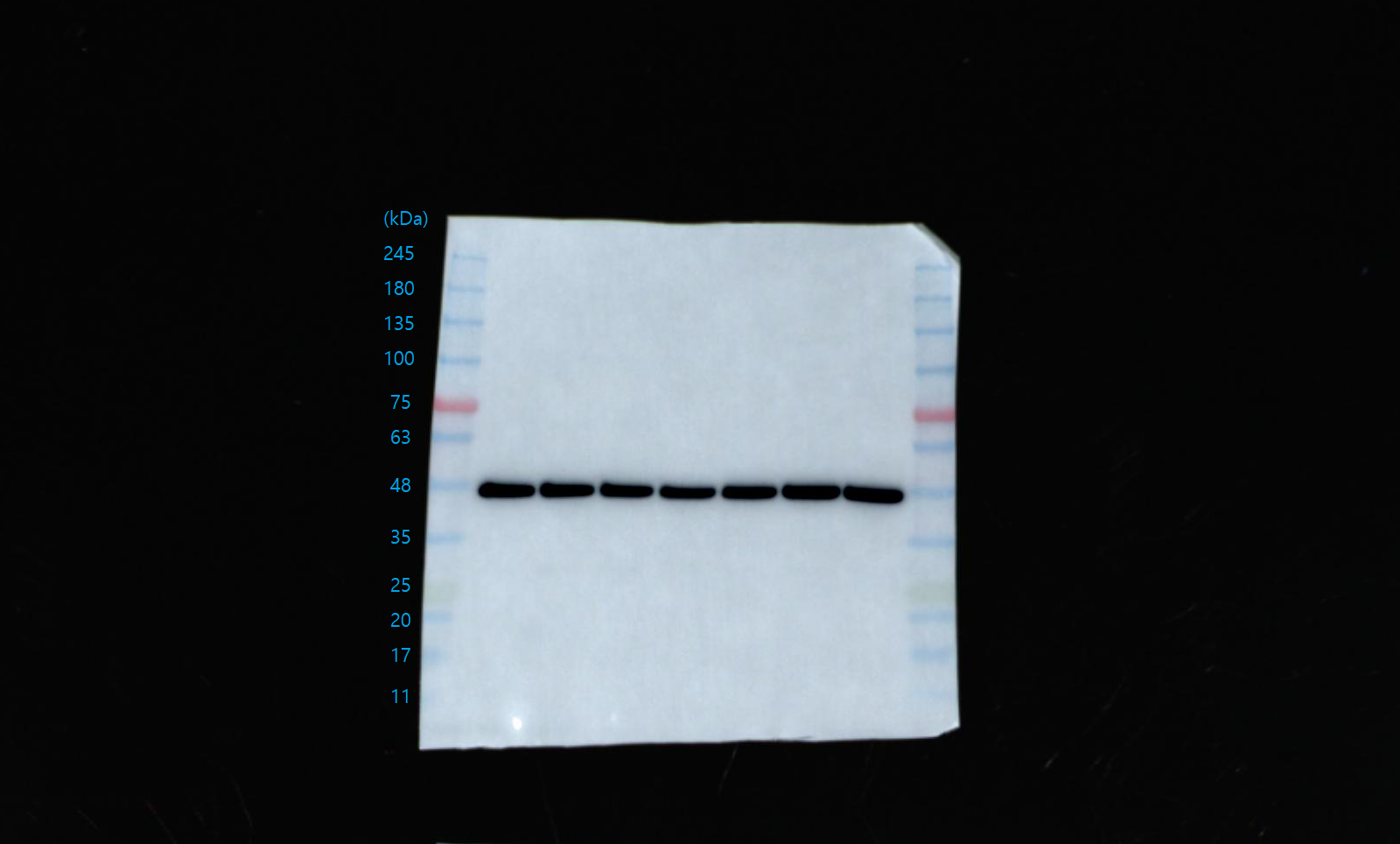

Supplement: Supplementary file 1 [file cancers-11-01849-s001.zip › Figure3(E)_western blot whole blot/PDC15_beta actin.tif]

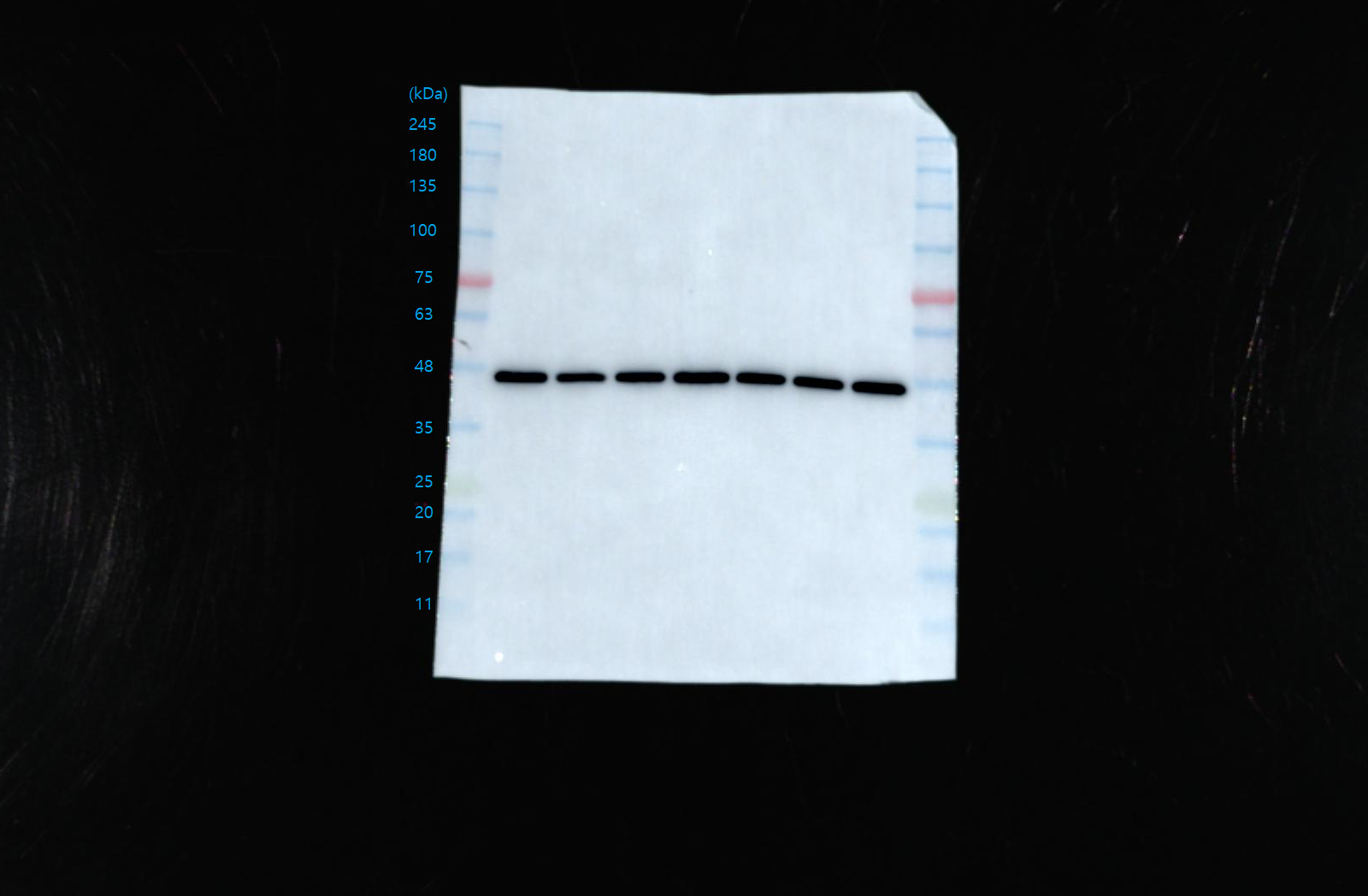

Supplement: Supplementary file 1 [file cancers-11-01849-s001.zip › Figure3(E)_western blot whole blot/PDC1_beta actin.tif]

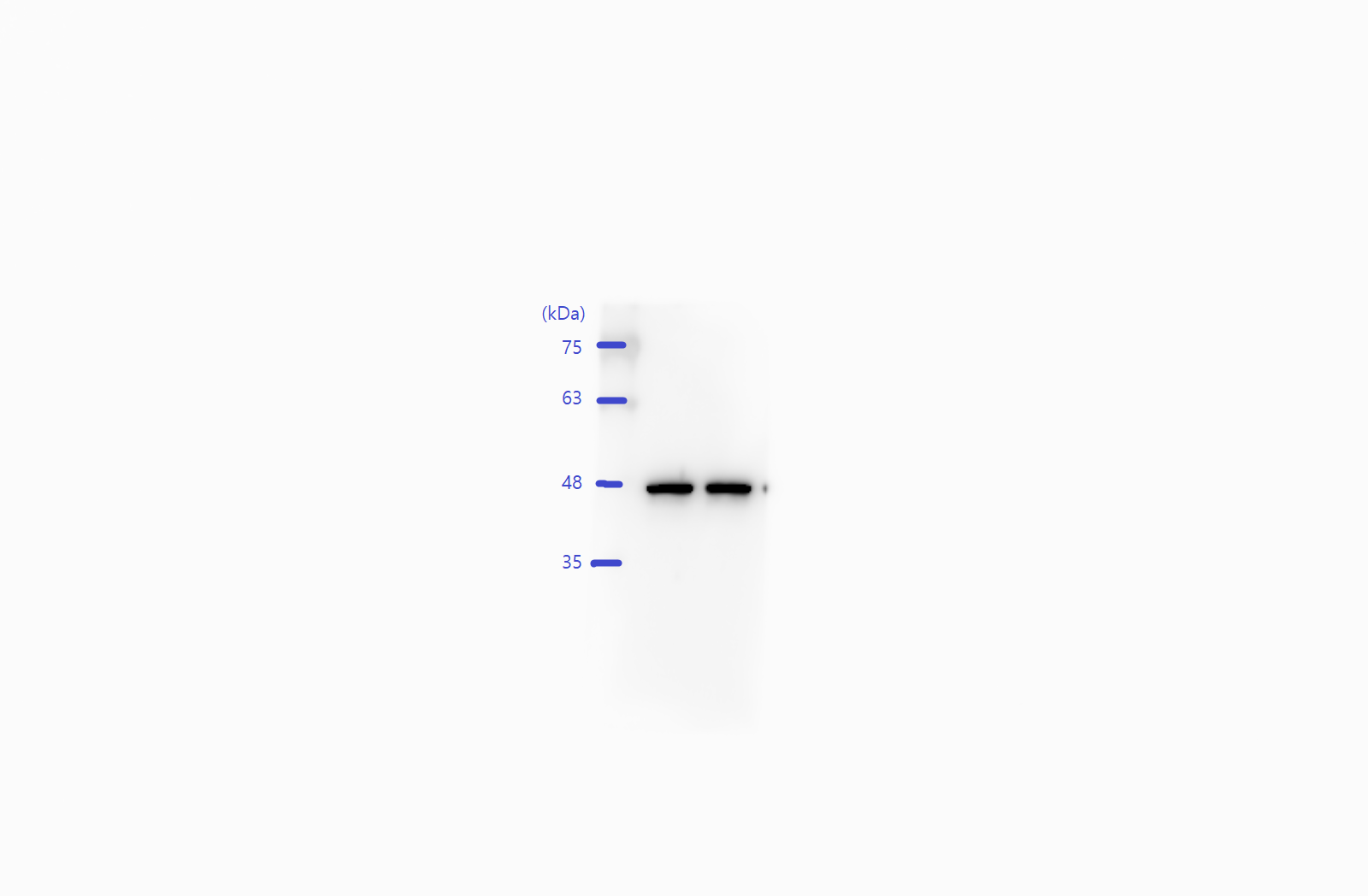

Supplement: Supplementary file 1 [file cancers-11-01849-s001.zip › Figure5(B)_western blot whole blot/PDC1_beta actin.tif]

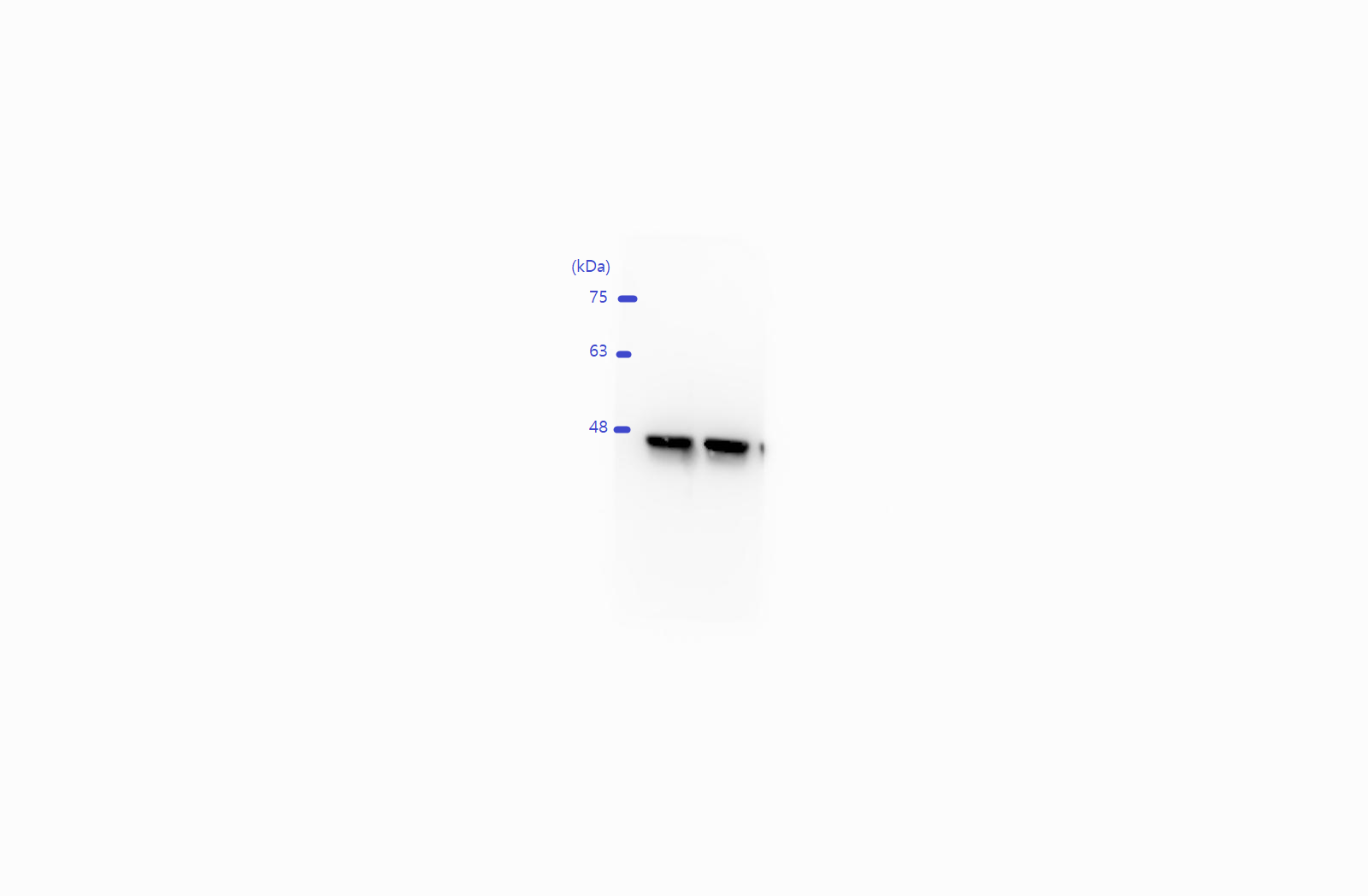

Supplement: Supplementary file 1 [file cancers-11-01849-s001.zip › Figure5(B)_western blot whole blot/PDC15_beta actin.tif]

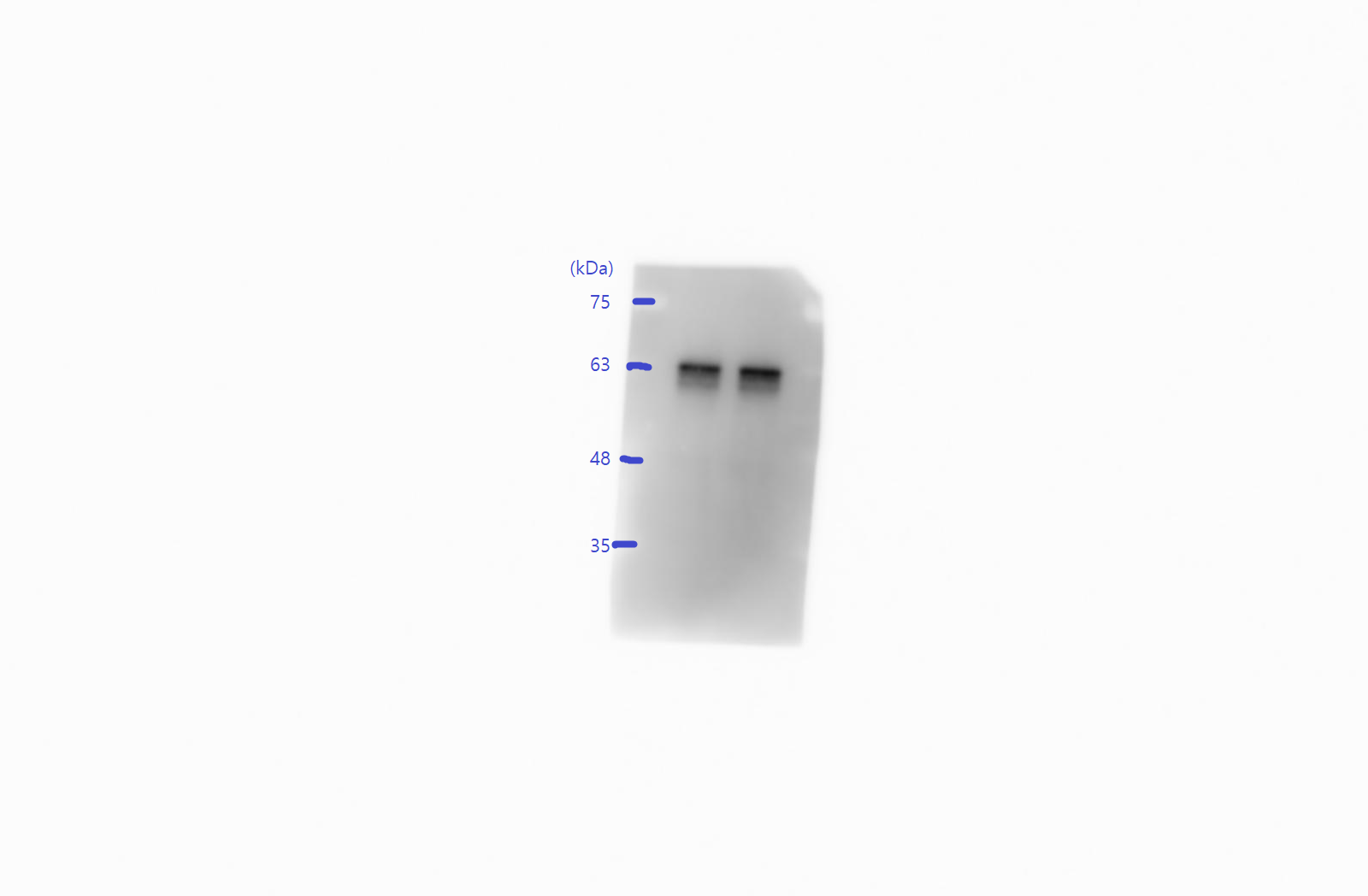

Supplement: Supplementary file 1 [file cancers-11-01849-s001.zip › Figure5(B)_western blot whole blot/PDC1_AKT.tif]

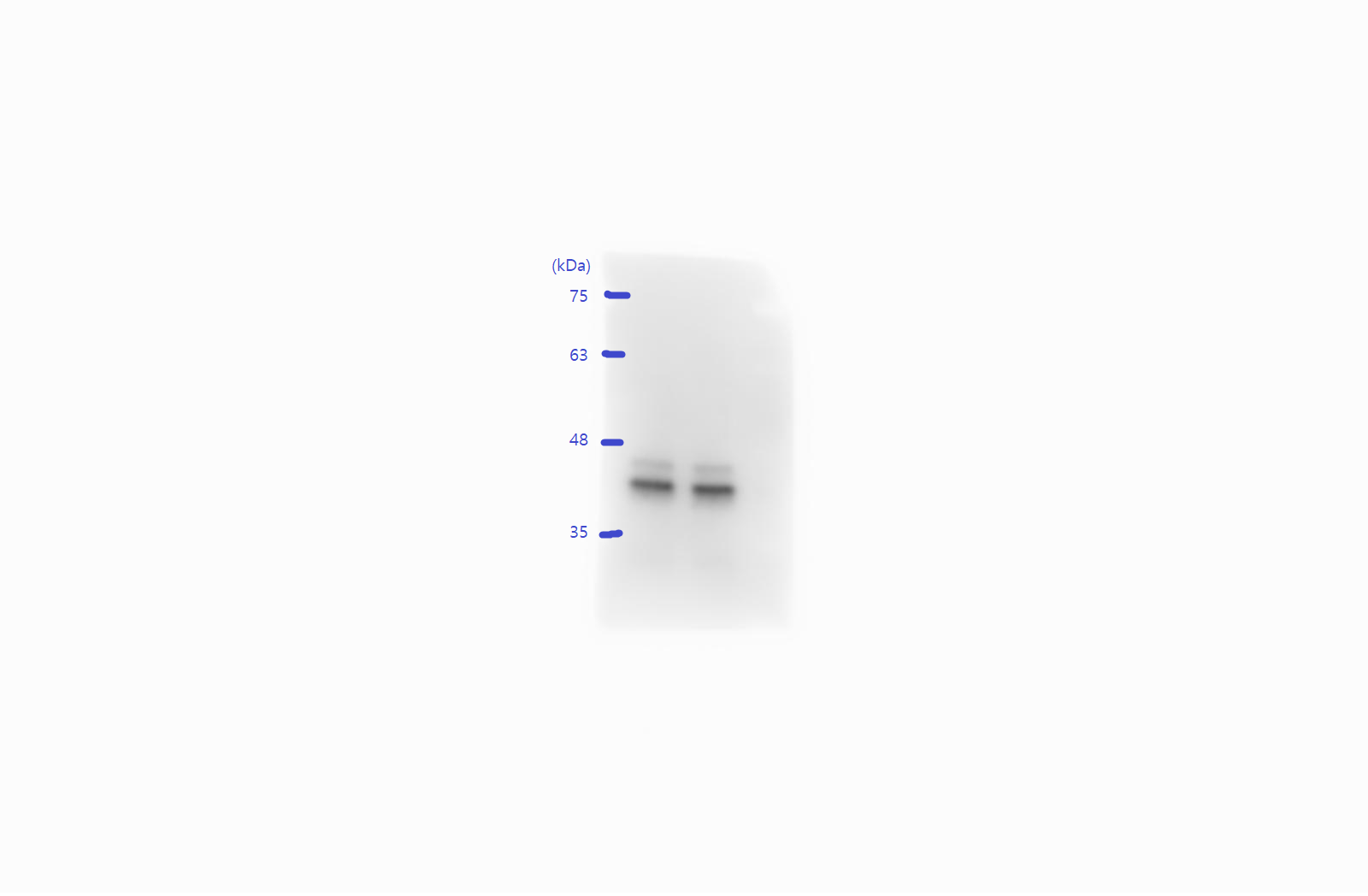

Supplement: Supplementary file 1 [file cancers-11-01849-s001.zip › Figure5(B)_western blot whole blot/PDC1_ERK.tif]

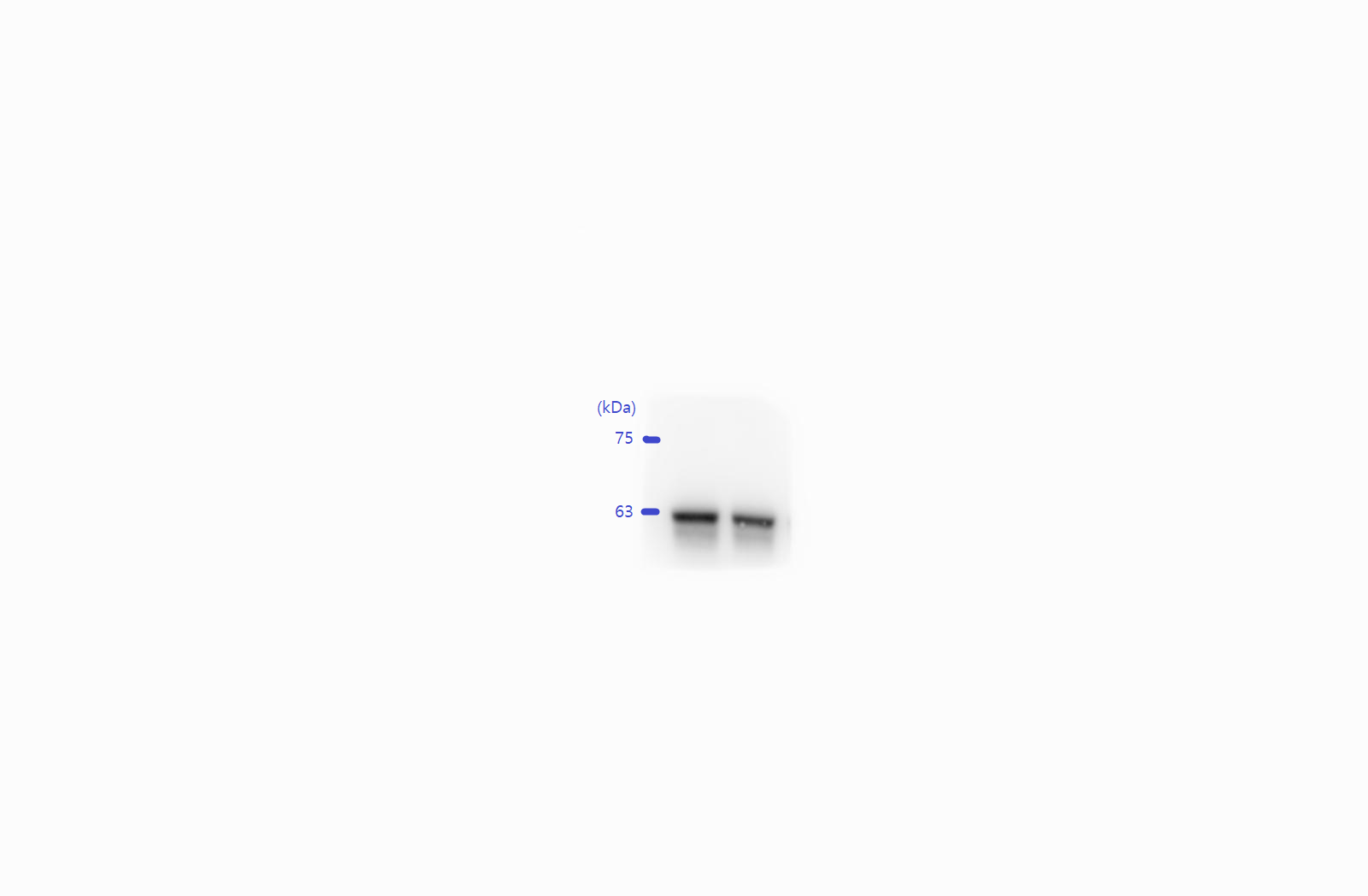

Supplement: Supplementary file 1 [file cancers-11-01849-s001.zip › Figure5(B)_western blot whole blot/PDC15_AKT.tif]

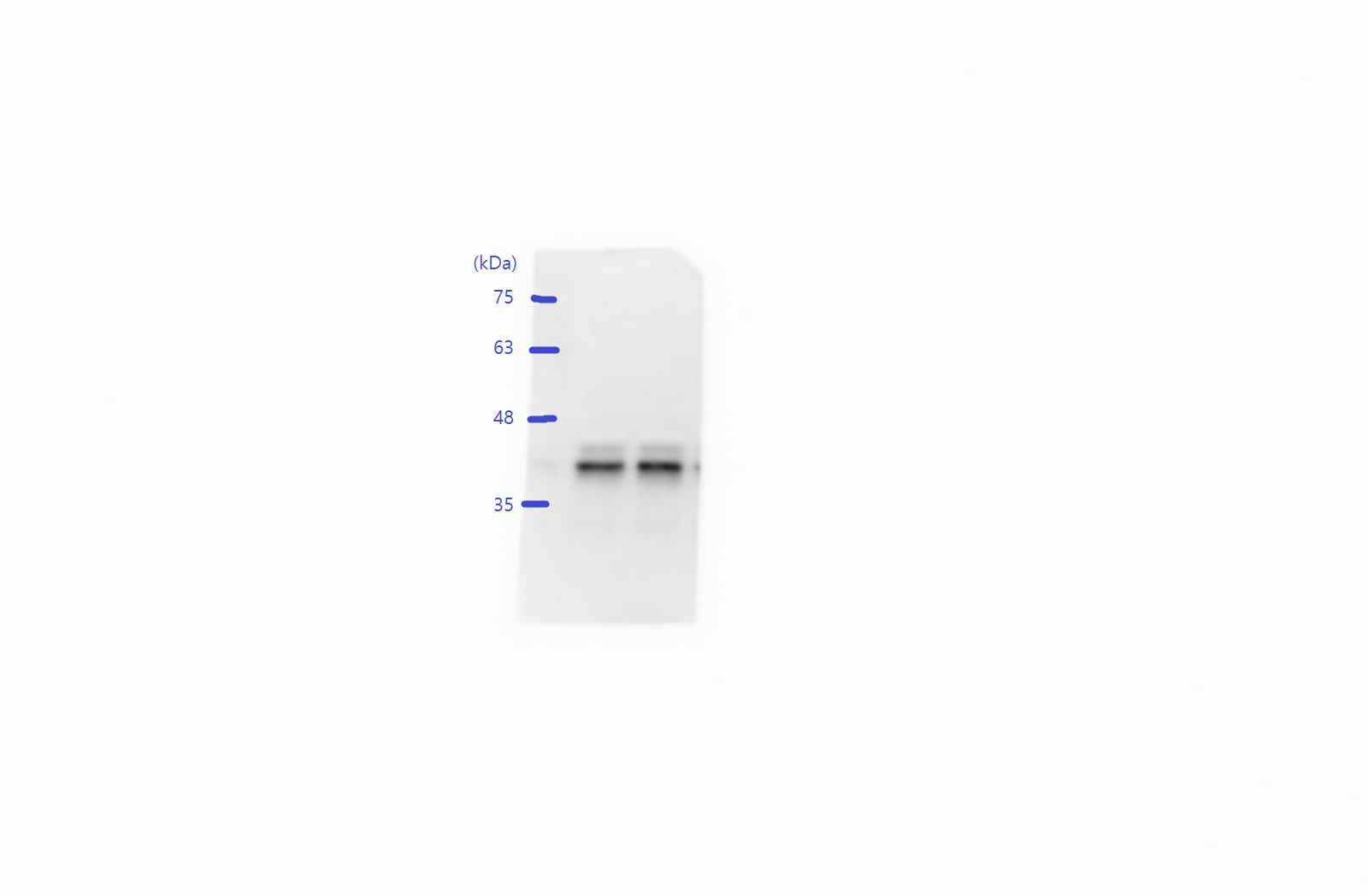

Supplement: Supplementary file 1 [file cancers-11-01849-s001.zip › Figure5(B)_western blot whole blot/PDC15_ERK.tif]

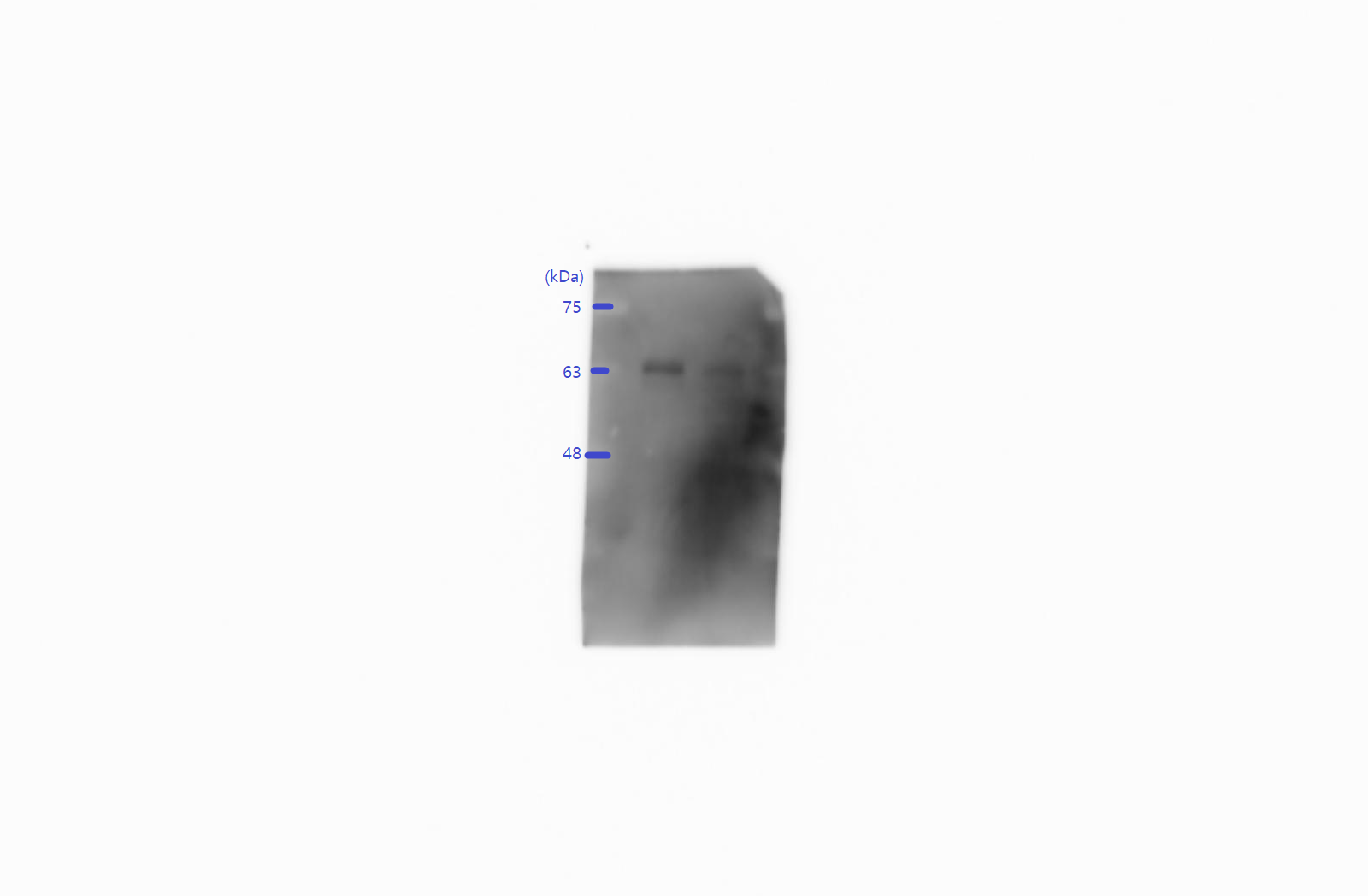

Supplement: Supplementary file 1 [file cancers-11-01849-s001.zip › Figure5(B)_western blot whole blot/PDC1_pAKT.tif]

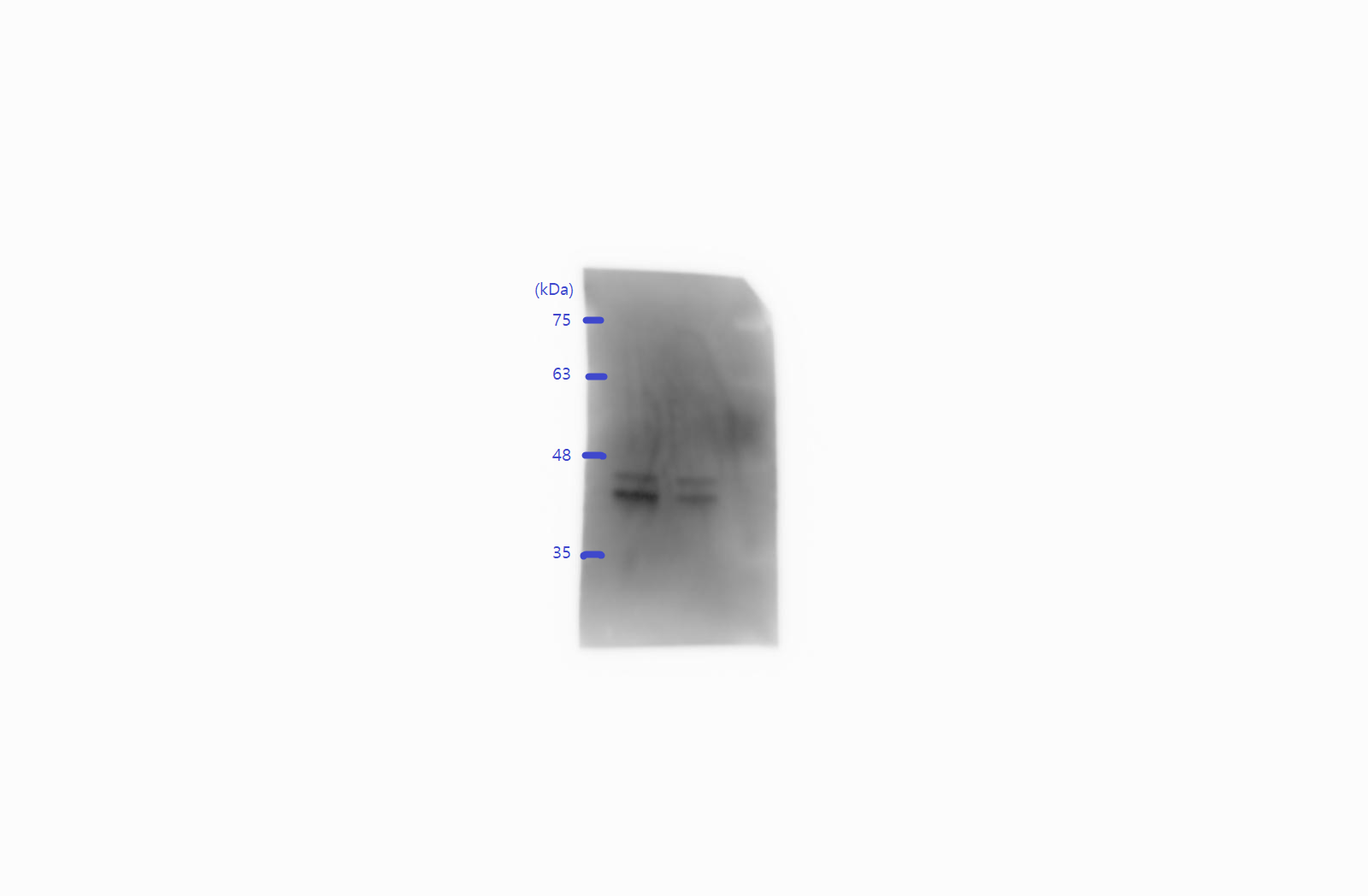

Supplement: Supplementary file 1 [file cancers-11-01849-s001.zip › Figure5(B)_western blot whole blot/PDC1_pERK.tif]

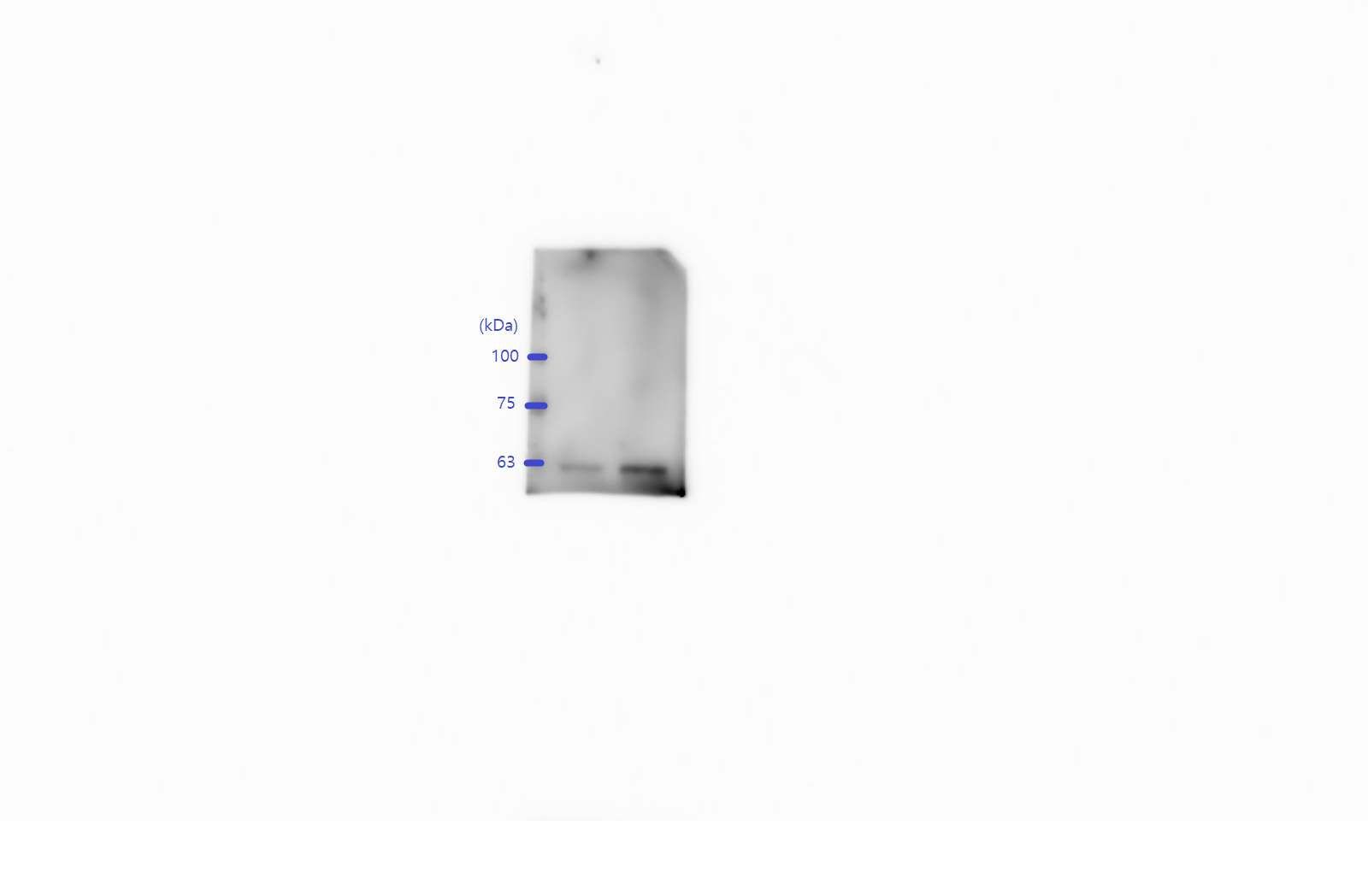

Supplement: Supplementary file 1 [file cancers-11-01849-s001.zip › Figure5(B)_western blot whole blot/PDC15_pAKT.tif]

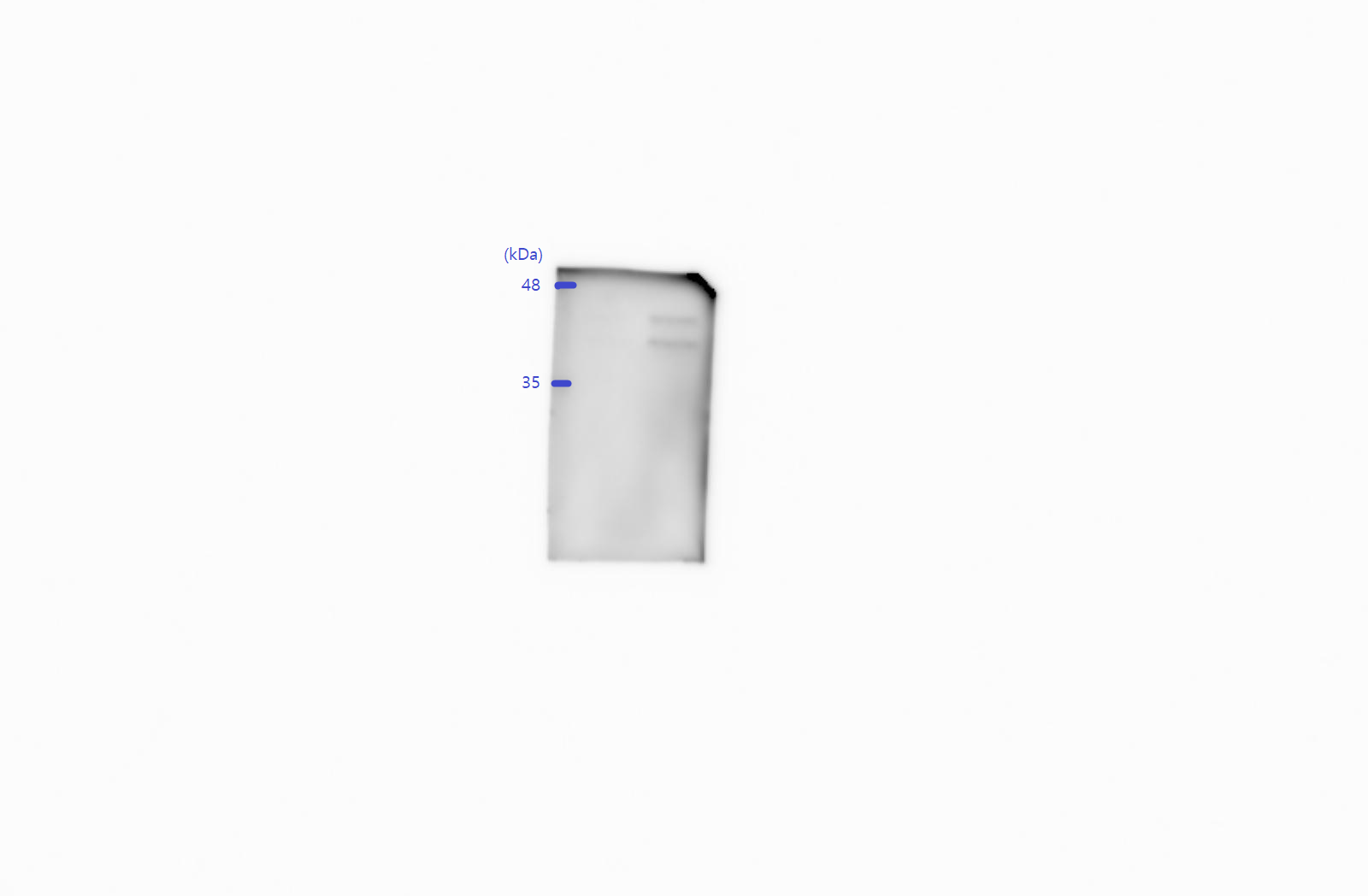

Supplement: Supplementary file 1 [file cancers-11-01849-s001.zip › Figure5(B)_western blot whole blot/PDC15_pERK.tif]

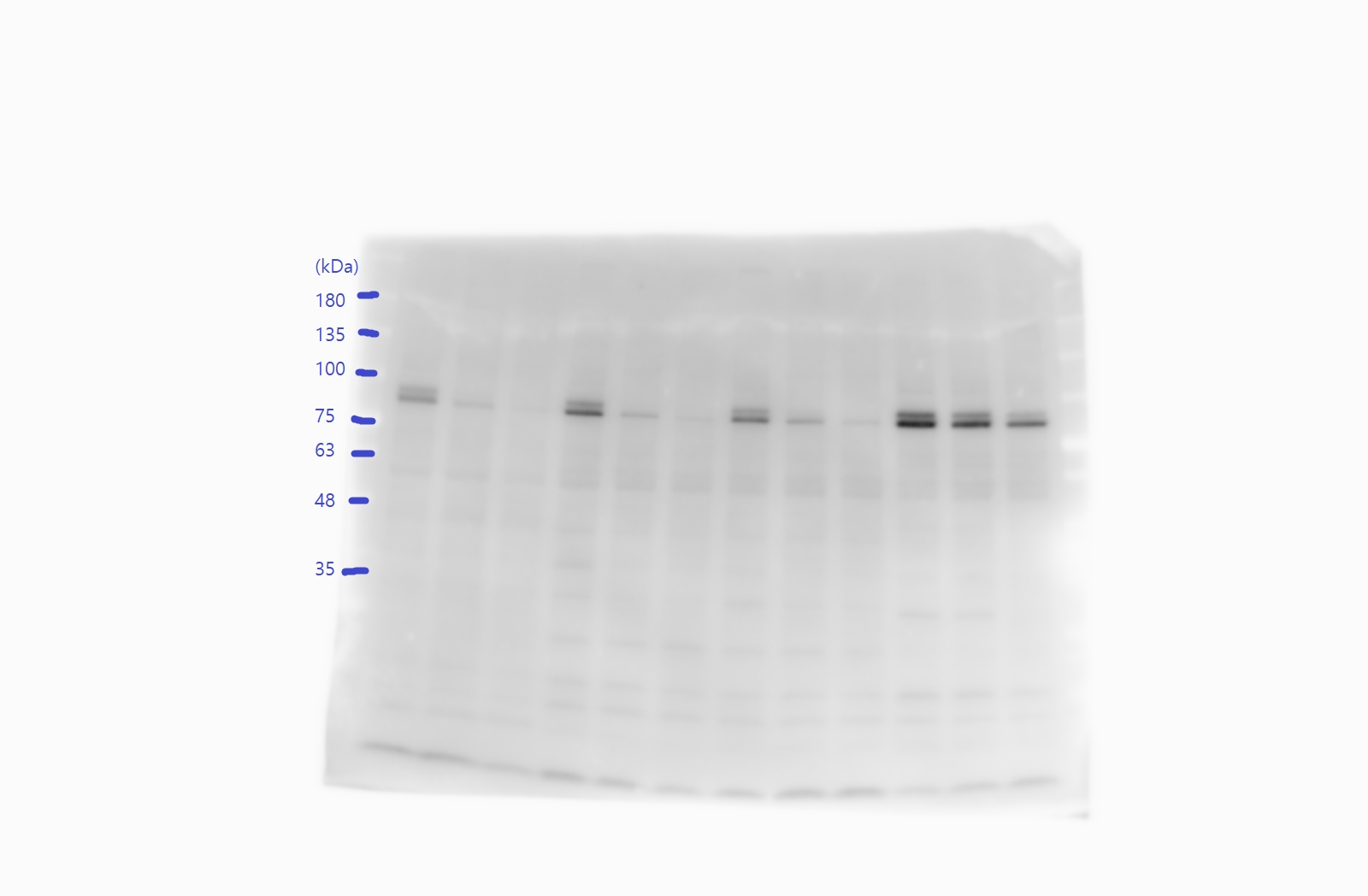

Supplement: Supplementary file 1 [file cancers-11-01849-s001.zip › Supplementary figure1_western blot whole blot/Nedd8 conjugated proteins.tif]

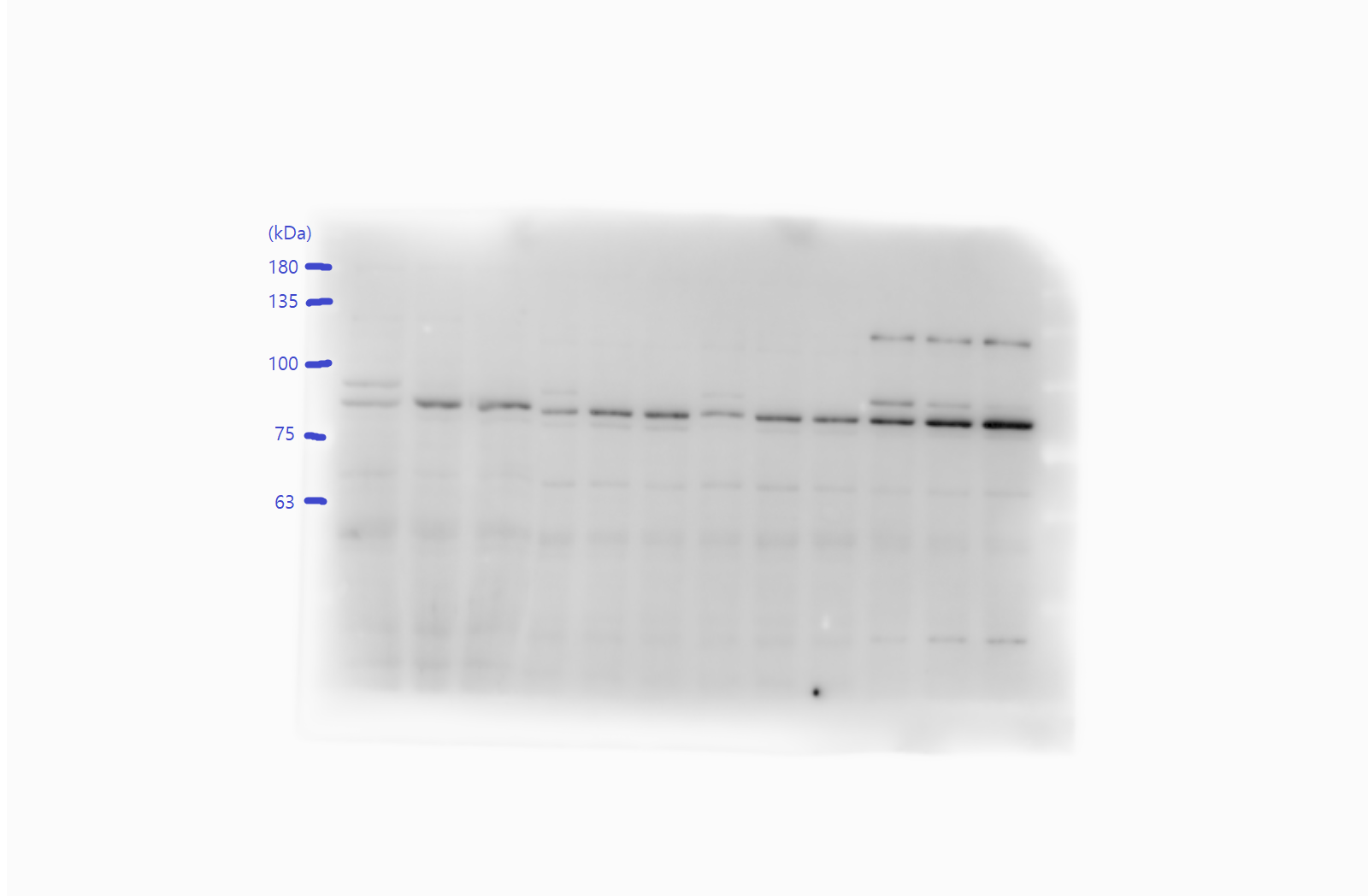

Supplement: Supplementary file 1 [file cancers-11-01849-s001.zip › Supplementary figure1_western blot whole blot/Cullin1.tif]

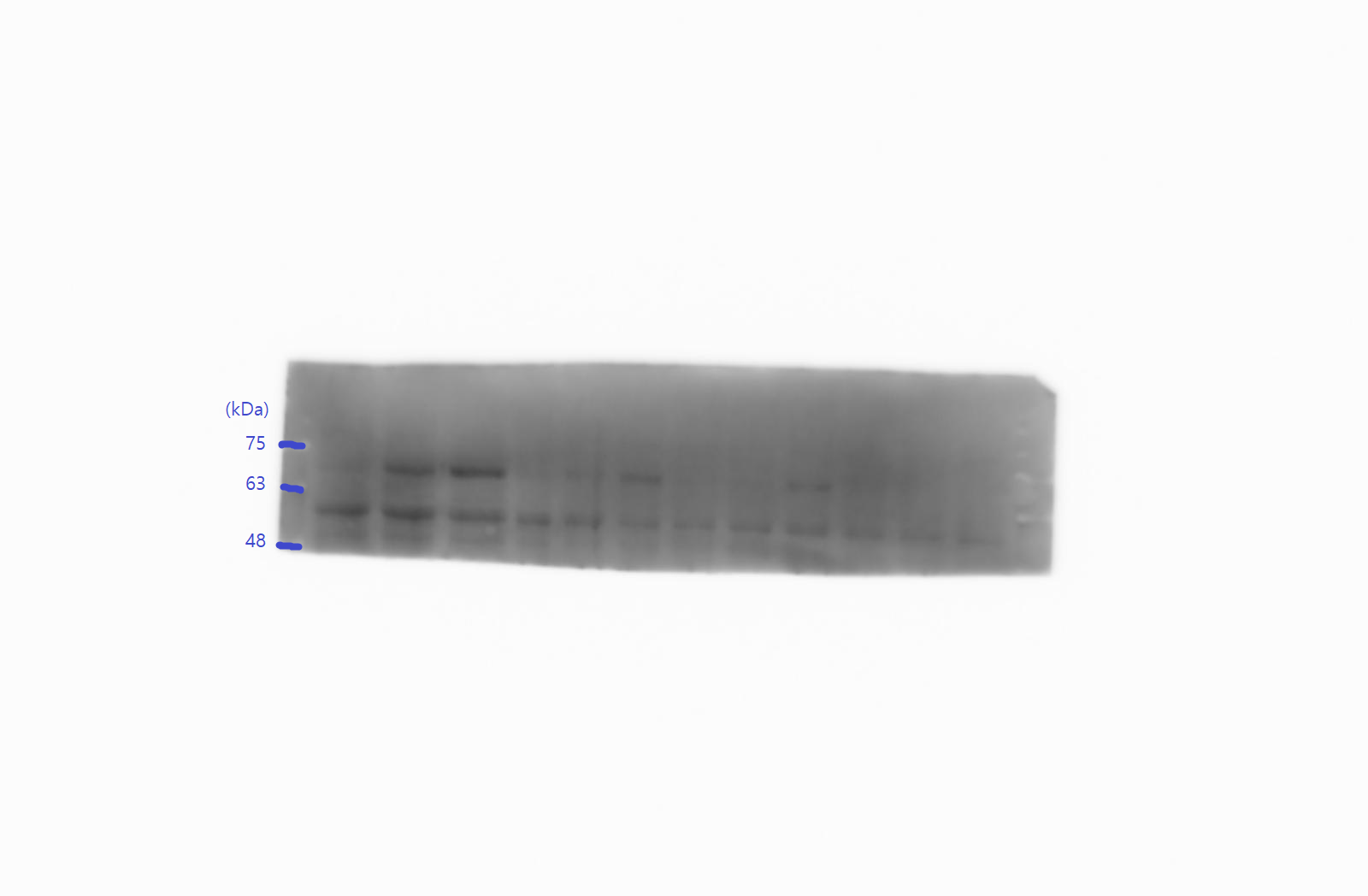

Supplement: Supplementary file 1 [file cancers-11-01849-s001.zip › Supplementary figure1_western blot whole blot/CDT1.tif]

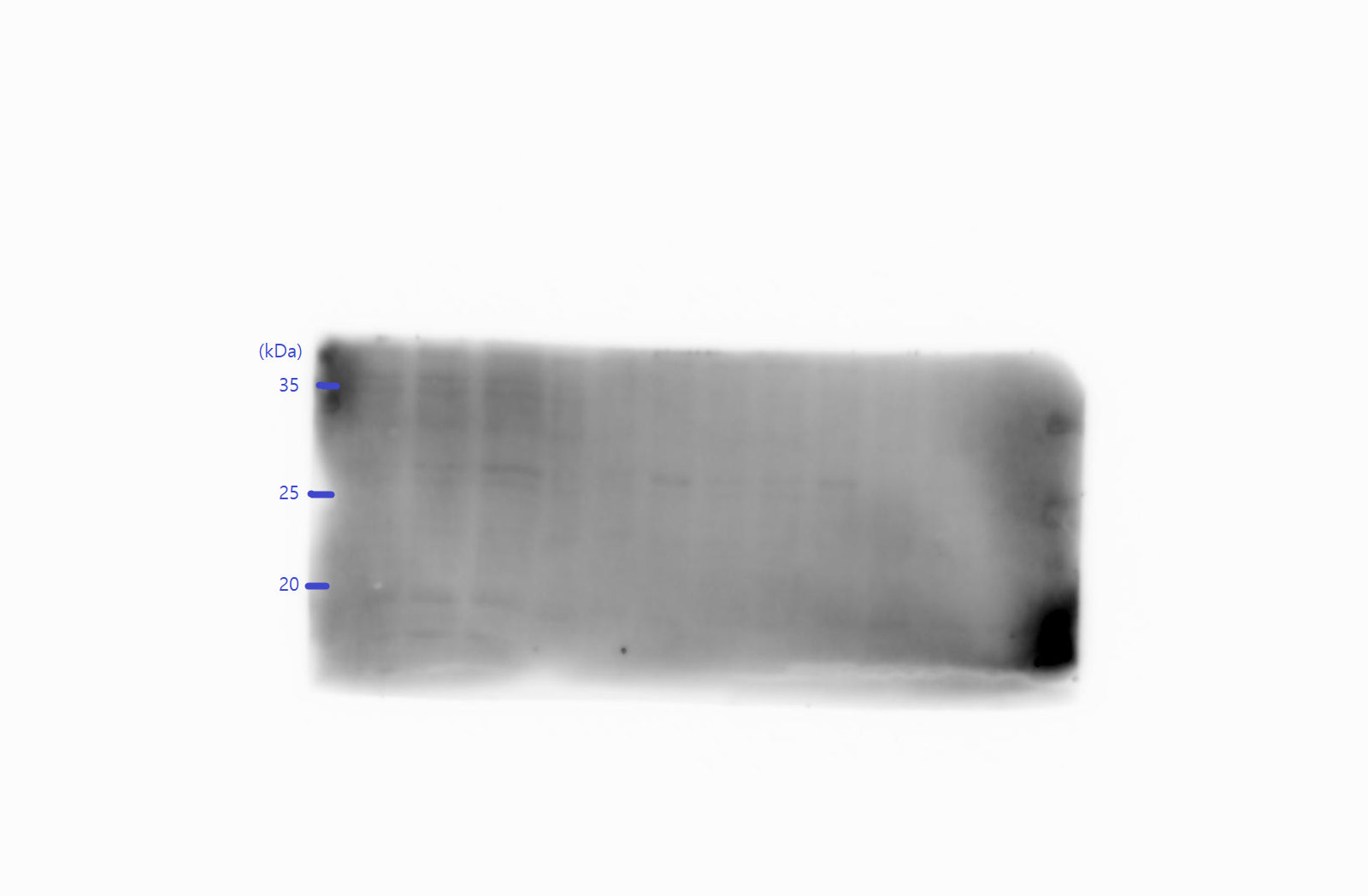

Supplement: Supplementary file 1 [file cancers-11-01849-s001.zip › Supplementary figure1_western blot whole blot/P27.tif]

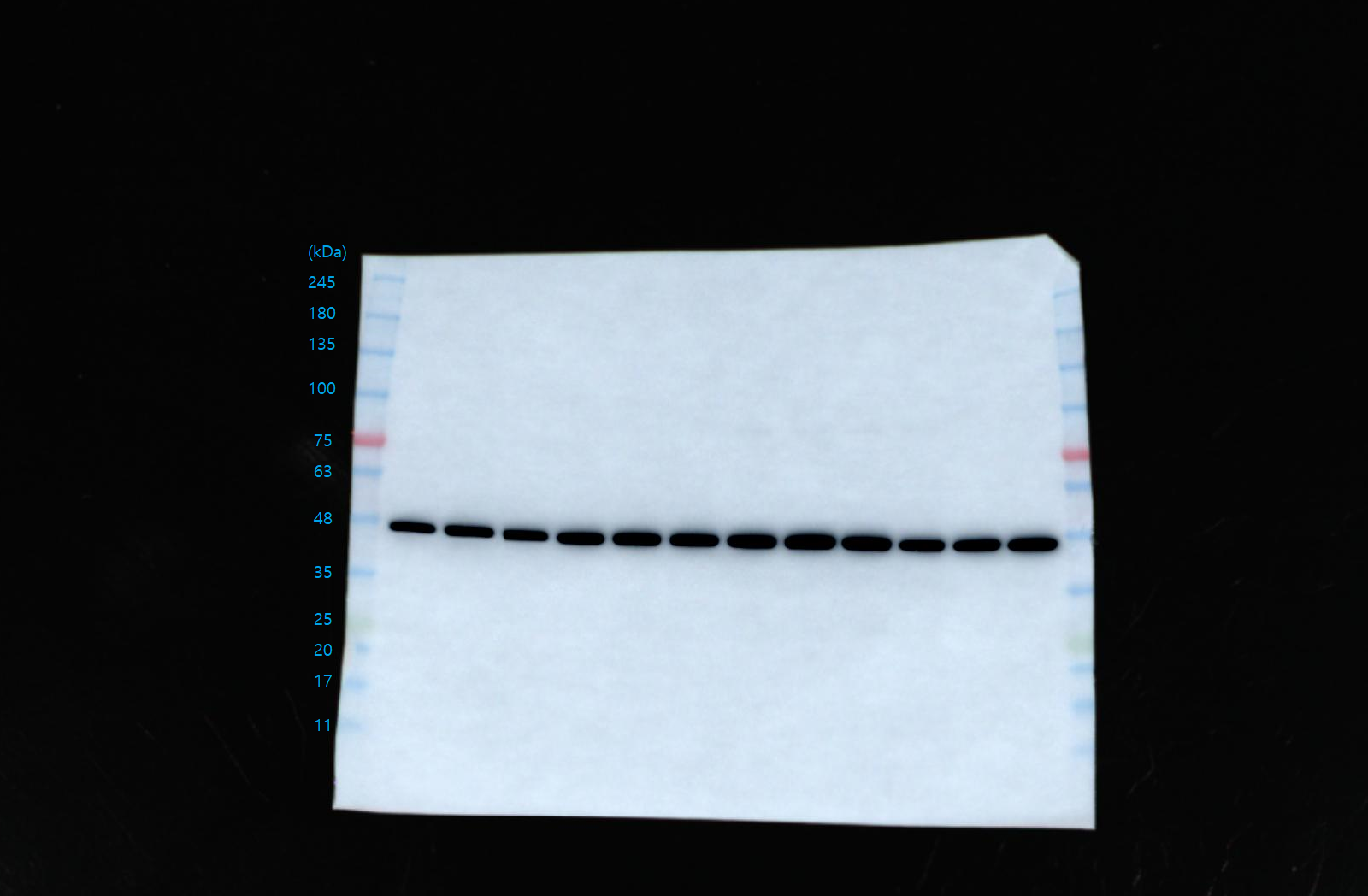

Supplement: Supplementary file 1 [file cancers-11-01849-s001.zip › Supplementary figure1_western blot whole blot/beta actin.tif]
